# Targeted disruption of *sp7* and *myostatin* with CRISPR-Cas9 results in severe bone defects and more muscular cells in common carp

**DOI:** 10.1038/srep22953

**Published:** 2016-03-15

**Authors:** Zhaomin Zhong, Pengfei Niu, Mingyong Wang, Guodong Huang, Shuhao Xu, Yi Sun, Xiaona Xu, Yi Hou, Xiaowen Sun, Yilin Yan, Han Wang

**Affiliations:** 1 Center for Circadian Clocks, Soochow University, Suzhou 215123, Jiangsu, China; 2 School of Biology & Basic Medical Sciences, Medical College, Soochow University, Suzhou 215123, Jiangsu, China; 3 Heilongjiang River Fisheries Research Institute of Chinese Academy of Fishery Sciences, Harbin, China; 4 Institute of Neuroscience, University of Oregon, Eugene, Oregon 97403, USA

## Abstract

The common carp (*Cyprinus carpio*) as one of the most important aquaculture fishes produces over 3 million metric tones annually, approximately 10% the annual production of the all farmed freshwater fish worldwide. However, the tetraploidy genome and long generation-time of the common carp have made its breeding and genetic studies extremely difficult. Here, TALEN and CRISPR-Cas9, two versatile genome-editing tools, are employed to target common carp bone-related genes *sp7, runx2, bmp2a, spp1, opg*, and muscle suppressor gene *mstn*. TALEN were shown to induce mutations in the target coding sites of *sp7, runx2, spp1* and *mstn*. With CRISPR-Cas9, the two common carp *sp7* genes, *sp7a* and *sp7b*, were mutated individually, all resulting in severe bone defects; while *mstnba* mutated fish have grown significantly more muscle cells. We also employed CRISPR-Cas9 to generate double mutant fish of *sp7a;mstnba* with high efficiencies in a single step. These results demonstrate that both TALEN and CRISPR-Cas9 are highly efficient tools for modifying the common carp genome, and open avenues for facilitating common carp genetic studies and breeding.

The common carp (*Cyprinus carpio*) as an omnivorous filter-feeder is cultured widely in more than 100 countries^1^, and its domesticated variant koi is the most popular outdoor ornamental fish due to its colorful scales and patterns^2^. Common carp as an important aquaculture species normally weighs more than 3 kg and can grow up to 1-meter long^3^. The annual production of common carp is approximately 3.7 million metric tons worthy of $5.31 billion worldwide^1^. Common carp also serves as a vertebrate model for extensive studies in ecology and evolution, environmental toxicology, physiology, nutrition, immunology, development, breeding and transgenics^4^,^5^,^6^. Even though common carp is economically important as a food source, its inter-muscular bones prevent it from being a favorable delicacy in some regions of the world^7^. Hence genetic modifications aiming to improve its qualities and economic value as well as to facilitate its utilities as a biological model are highly demanded. However, due to its relatively long maturation period of approximately 3–4 years^4^, and less frequent spawning of only once a year, genetic studies and breeding of common carp have been extremely difficult^2^.

With the recent report of the common carp genome draft sequences^1^ as well as other genetic and genomic resources^4^,^8^,^9^,^10^,^11^, the most pressing need for the common carp field is to develop its genome-editing tools. To this end, we set to apply both TALEN (Transcription Activator-Like Effector Nucleases)^12^ and CRISPR (Clustered, regularly interspaced, short palindromic repeats (CRISPR)-CRISPR-associated (Cas) systems)^13^ in common carp. TALEN and CRISPR-Cas9 are two engineered site-specific genome-editing systems, each composed of a DNA-recognizing/binding part and a DNA-cleavage part^14^,^15^. In the TALEN system, the two TALE domains are the DNA-recognizing/binding part and the two FokI domains are the DNA-cleavage part^12^, whereas in the CRISPR-Cas9 system, the single guide-RNA (gRNA) is responsible for DNA-recognizing/binding and the Cas9 endonuclease acts to cleave DNAs^13^. A pair of TALENs or one gRNA with the Cas9 protein can cause site-specific DNA double-strand breaks (DSBs) that induce the endogenous nonhomologous end joining (NHEJ) DNA repair pathway to result in indel mutations in targeted genes of human cells and many species including rat, mouse, zebrafish, cow and plants^14^,^16^,^17^,^18^,^19^,^20^,^21^,^22^,^23^,^24^,^25^ as well as in numerous fields such as basic research, clinical treatment, agriculture and animal husbandry^20^,^23^,^26^,^27^. Moreover, it appears that CRISPR-Cas9 has advantages of ease to carry out, high efficiencies and low cost over TALEN^13^,^15^,^25^,^28^,^29^,^30^,^31^, and particularly CRISPR-Cas9 allows for mutagenizing multiple genes in the stem cells or zygotes, and generating bialleic mutants with clear phenotypes in the F_0_ generation for studying gene functions without crossing animals for several generations^24^,^32^,^33^,^34^. However, both methods have not been applied in common carp to date.

Here, we have employed both TALEN and CRISPR-Cas9 techniques to modify genes involved in bone formation such as *sp7*, a zinc-finger-containing transcription factor expressed in osteoblasts^35^, which can activate pre-osteoblast cells to differentiate into mature osteoblast cells and osteocytes^36^,^37^. In addition, we have used both techniques to modify other genes involved in bone formation including *runx2* (runt-related transcription factor 2), an essential transcription factor to regulate osteoblast differentiation and bone development^38^; *spp1* (secreted phosphoprotein 1), a late osteoblast specific marker involved in bone mineralization^39^,^40^; *opg* (osteoprotegerin), a bone-protecting molecule participated in impairing the osteoclast formation^41^; and *bmp2* (encoding for bone morphogenetic protein 2), promoting expression of *runx2* and *sp7* and then inducing expression of *spp1*, *osteocalcin* and other osteogenic genes^42^. We also have used both systems to modify *mstnba*, a member of the transforming growth factor-β superfamily and a negative regulator of the skeletal muscle growth^43^,^44^. *Mstn* knockout mice displayed 2 to 3 fold increase in both myofiber size and number of muscular cells compared to the wild-type littermates^45^,^46^. Our results showed that *mstnba*-CRISPR and *sp7a*-CRISPR mutated common carps display defects in muscle or bone. We also generated double mutants of *mstnba*;*sp7a* in common carp with high efficiencies. Together, these results demonstrate that both TALEN and CRISPR-Cas9 systems are effective genome-editing tools for common carp genetic studies and breeding.

## Results

### Design of TALEN and CRISPR-Cas9 target sites

We designed TALENs and CRISPR-Cas9/gRNAs targeting genes involved in bone and muscle development. In the bone formation pathway, *bmp2a* is an up-stream gene, *runx2* and *sp7* are mid-stream genes, and *spp1* is down-stream genes^47^. In addition, *opg* acts to inhibit osteoclast formation^41^. *mstn* is involved in muscle formation^48^. Among the selected genes, *sp7a, runx2 and mstnba* were edited using both TALEN and CRISPR-Cas9 systems to examine efficiencies of these two methods.

The cDNA sequences of zebrafish orthologs in ENSEMBL or NCBI were used to interrogate the common carp genome database (http://www.carpbase.org/login.php)^1^. Common carp DNA sequences selected were then blasted against the common carp protein database (http://www.carpbase.org/login.php) to predict the exon-intron structure. In addition, the selected target sites were blasted back against the common carp genome with reciprocal top blast hit for the target regions to minimize off-target effects.

One pair of TALENs each for *runx2*, *sp7a*, *mstnba* and *spp1a* was designed. Since the spacer length is very important to mutation efficiencies^49^, 3 pairs of the TALENs designed for *runx2a*, *sp7a* and *mstnba* were longer than 22 bp and 1 pair of TALENs for *spp1a* was shorter than 22 bp for comparison. In addition, there were restriction endonuclease sites located in the spacers of *runx2* and *mstnba* (Supplementary Figs S1 and S2).

For CRISPR-Cas9 targeting, six genes including *sp7a*, *sp7b*, *mstnba*, *runx2*, *opga* and *bmp2ab* were selected. Most of the gRNAs were designed to target the first exon of the corresponding genes, except for *runx2* and *sp7b*, whose gRNAs target exon 3 and exon 2, respectively (Supplementary Figs S4 and S7). All the target sites include 20-bp sequence and PAM sequence (protospacer adjacent motif NGG). The targeted regions for all these genes have restriction endonucleases sites located either near the PAM sequence or on the PAM for subsequent genotyping (Supplementary Figs S4–S7).

### Evaluate mutagenesis efficiencies of TALEN and CRISPR-Cas9 of common carp genes using zebrafish embryos

**Figure 1:**
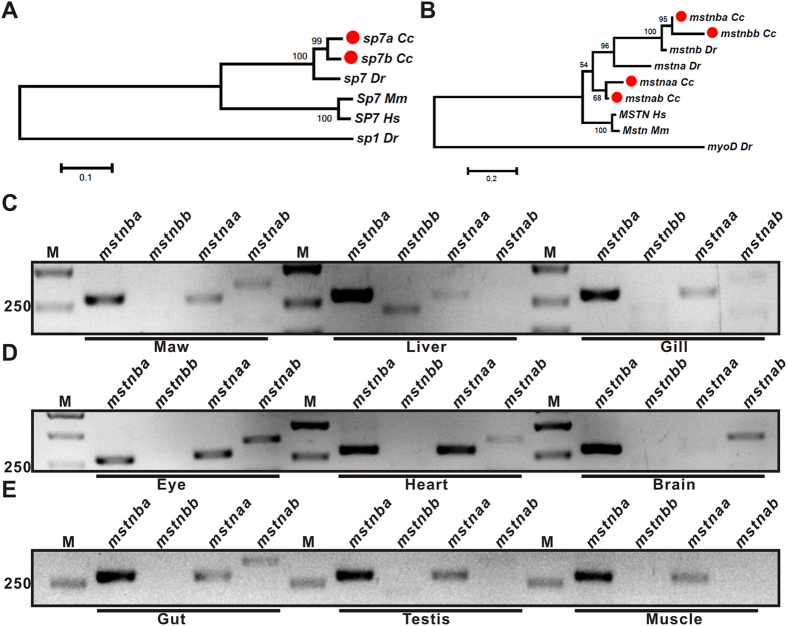
Phylogenetic analysis of SP7 and MSTN proteins and expression of common carp *mstn* genes. Phylogenetic trees of SP7 proteins (**A**) and MSTN proteins (**B**) were constructed by Neighbor-joining (NJ) using MEGA6.06^64^ with the maximum-likelihood method. The number of bootstrap replication was 1000. The numbers indicate the support value. *Dr*, *Danio rerio*; *Hs*, *Homo sapiens*; *Mm*, *Mus musculus*; and *Cc*, *Cyprinus carpio*. The common carp proteins are labeled in solid red circle. Zebrafish Sp1 and MyoD proteins serve as outgroups. Expression of *mstn* genes in common carp tissues/organs: maw, liver and gill (**C**), eye, heart and brain (**D**), and gut, testis and muscle (**E**), as shown by qRT-PCR. M, marker. Electrophoretic results were cropped from the original images shown in Supplementary Fig. S13A–C.

Phylogenetic analysis showed that common carp have two *sp7* genes and four *mstn* genes, while zebrafish have only one *sp7* gene and two *mstn* genes (Fig. 1A,B), consistent with tetraploidy nature of common carp genome^10^. RT-PCR analysis showed that *mstnba* is expressed highly in all the tissues/organs including the maw, liver, gill, eye, heart, brain, gut, testis and muscle, while the other three *mstn* genes are expressed only in some of these tissues/organs (Fig. 1C–E). We therefore selected *mstnba* in the following experiments.

**Figure 2:**
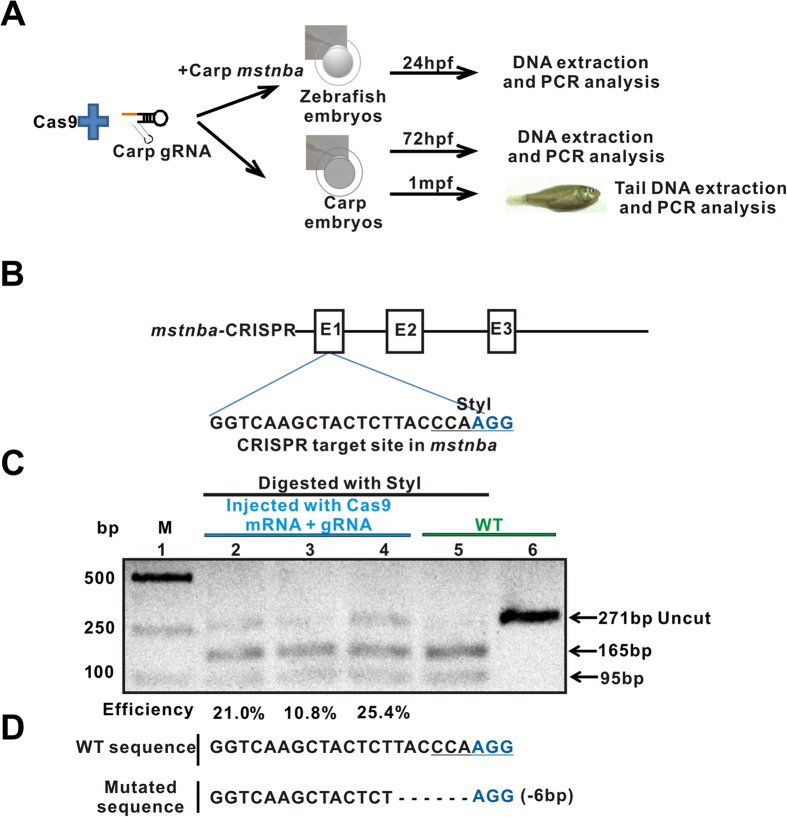
CRISPR-Cas9-induced mutagenesis efficiencies of common carp genes evaluated in zebrafish embryos. (**A**) Flowchart of the CRISPR-Cas9 method. Up panel: The plasmid of common carp gene was co-microinjected with Cas9 mRNA and gRNA into one- or two-cell zebrafish embryos. DNA fragments were PCR amplified from the zebrafish embryos at 24 hpf (hours postfertilization); Middle panel: The common carp gRNA was co-microinjected with Cas9 mRNA into one- or two- cell common carp embryos. DNA fragments were amplified from the common carp embryos at 72 hpf; and Bottom panel: Fin-clipped DNAs from one-month-old carp injected with *msnba-* and *sp7a-*CRISPR-Cas9 were extracted and PCR amplified. (**B**) Schematic of the Cas9-gRNA-targeted site in common carp *mstnba*. The protospacer-adjacent motif (PAM) sequence is labeled in blue. The StyI restriction site at the target region is underlined. E: exon. (**C**) Enzymatic digestion analysis of Cas9-mediated cleavage at the protospacer in common carp *mstnba* in zebrafish embryos. Lane 1, marker; lanes 2–4 (below the blue line), 25, 50, 100 pg of *mstnba g*RNA for injection; lanes 5–6, wild-type control (below the green line); *mstnba* PCR products were digested with StyI (lanes 2–5, below the black line); lane 6, wild-type control, *mstnba* PCR products. Uncut (271 bp) and cut (165 bp and 96 bp) PCR bands are indicated. WT, wild type; M, marker. Electrophoretic results were cropped from the original images shown in Supplementary Fig. S13D. (**D**) A 6-bp deletion in the CRISPR-Cas9-targeted site of *msnba*, revealed by DNA sequencing analysis of the uncleaved PCR fragments.

Because of the ease of zebrafish embryos to work with, we first used them to evaluate mutagenesis efficiencies of TALENs and CRISPR-Cas9/gRNAs of common carp (Fig. 2A). Specifically, we examined common carp *runx2* and *sp7a* TALENs and common carp *mstnba* and *sp7a* CRISPR-Cas9/gRNAs in zebrafish embryos. For TALEN, 250 ng/ul capped mRNAs of each arm plus 50 ng/ul purified plasmids carrying the common carp genomic DNA fragments containing the *runx2*- or *sp7a*- targeted sites were microinjected to one-cell stage zebrafish embryos, respectively. No toxicity was observed for zebrafish embryos at these concentrations of TALENs and purified plasmids (Supplementary Table S1). The common carp DNA fragments containing *runx2*- or *sp7a*-targeted sites were PCR amplified from the microinjected zebrafish embryos at 24 hpf. Sequencing analyses revealed that TALENs induced a 6-bp insertion mutation in *runx2*-targeted site and a 16-bp deletion in *sp7a*-targeted sites (Supplementary Table S2).

For *mstnba*-CRISPR-Cas9, 25, 50 and 100 ng/ul gRNAs and 300 ng/ul capped Cas9 RNA plus 100 ng/ul purified common carp plasmid containing the targeted site were microinjected into one-cell zebrafish embryos. No toxicity was observed for zebrafish embryos at these concentrations of Cas9/gRNAs and purified plasmids (Supplementary Table S1). Then PCR products amplified from the microinjected zebrafish embryos at 24 hpf were digested with the StyI enzyme (Fig. 2A,B). ImageJ analysis revealed that mutation frequencies average approximately 19% (Fig. 2C). Sequencing analysis identified a 6-bp deletion mutation (Fig. 2D). For *sp7a*-CRISPR-Cas9 injected zebrafish embryos, digestion of the PCR product with the HinfI enzyme estimated that the mutation frequency was approximately 2.5% (Supplementary Table S2). Taken together, these results demonstrate that TALEN or CRISPR-Cas9-induced common carp mutagenesis activities can be conveniently evaluated in zebrafish embryos^50^.

### Mutagenesis efficiencies of TALEN and CRISPR-Cas9 in common carp embryos

**Figure 3:**
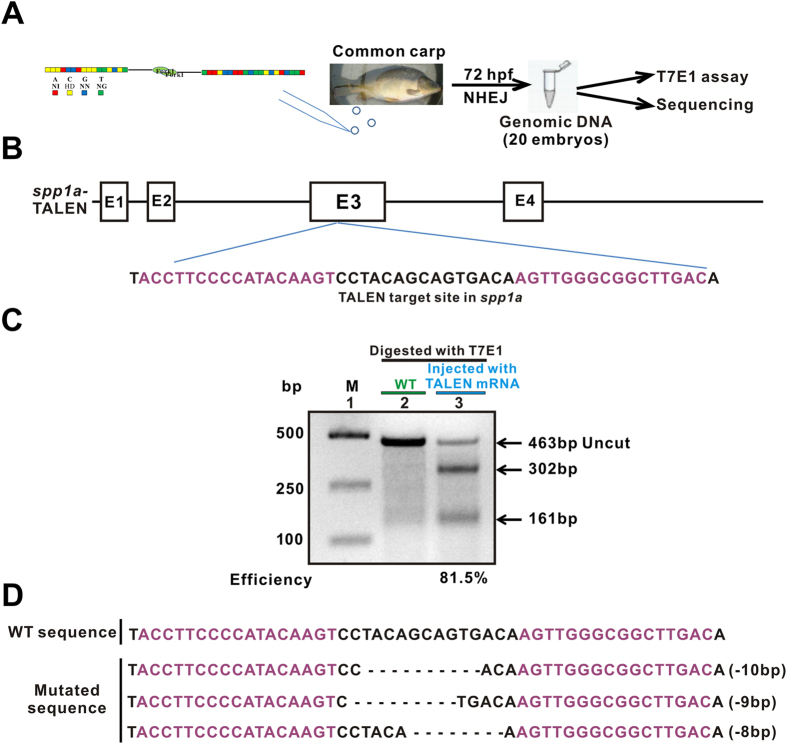
TALEN-induced mutagenesis efficiencies of common carp *spp1a* evaluated in common carp embryos. (**A**) Flowchart of the TALEN method. TALEN mRNAs of carp *spp1a* were microinjected into one- or two-cell common carp embryos. The targeted fragment was PCR amplified from the common carp embryos at 72 hpf and then digested with T7E1 endonuclease or subcloned for sequencing analysis. (**B**) Diagram of the TALEN-targeted site in common carp *spp1a*. The left and right arms of *spp1a* TALEN are highlighted in purple. E, exon. (**C**) T7E1 endonuclease analysis of TALEN-mediated cleavage at common carp *spp1a*. Lane 1, marker; lane 2, wild-type control (below the green line); lane 3, injected group at a concentration of 250 pg for each *spp1a* TALEN arm (below the blue line). The *spp1a* PCR products of wild-type and injected carps were digested with T7E1 endonuclease (below the black line). Uncut (463 bp) and cut bands (302 bp and 161 bp) are indicated. 250 pg of *spp1a* TALEN mRNAs were injected into one- or two-cell common carp embryos. Mutagenesis efficiency was estimated to be 81.5%. WT, wild type; M, marker. Electrophoretic results were cropped from the original images shown in Supplementary Fig. S13E. (**D**) Three types of deletion mutations, −10 bp, −9 bp, and −8 bp in the *spp1a* TALEN target site, revealed by DNA sequencing analysis. Mutations were detected in 3 out of the 4 sequenced alleles.

TALEN pairs each for *runx2*, *sp7a*, *mstnba* and *spp1a* were microinjected into the one-cell common carp embryos at a concentration of 250 ng/ul per arm, respectively (Fig. 3A,B). DNA fragments harboring targeted sites were PCR amplified from approximately 20 embryos at 72 hpf from each injected group, and digested with T7E1 enzyme. ImageJ analysis revealed that mutagenesis frequencies are 15.2% for *runx2*, 36.8% for *sp7a*, 29.1% for *mstnba* and 81.5% for *spp1a* (Fig. 3C and Supplementary Table S3). Digestion of the PCR products containing the *runx2*-targeting site or the *mstnba* -targeting site with HinfI or XbaI also estimated that mutation efficiencies are 1.23% for *runx2*, and 13.2% for *mstnba* (Supplementary Figs S1, S2 and Supplementary Table S3). Alternatively, these PCR-amplified fragments were cloned into the PMD-19T vector, respectively. DNA sequencing analysis showed that mutation efficiencies were 5% (1 out of the 20 single clones) for *runx2*, 20% (4 out of the 20 clones) for *sp7a*, 27.3% (3 out of the 11 clones) for *mstnba* and 75% (3 out of the 4 clones) for *spp1a* (Fig. 3D; Supplementary Figs S1–S3 and Supplementary Table S3), consistent with the enzymatic analyses. For TALEN-induced mutation efficiencies in common carp, the shorter the spacer lengths lead to the higher mutation efficiencies (Supplementary Table S4), as reported in other species previously^49^.

**Figure 4:**
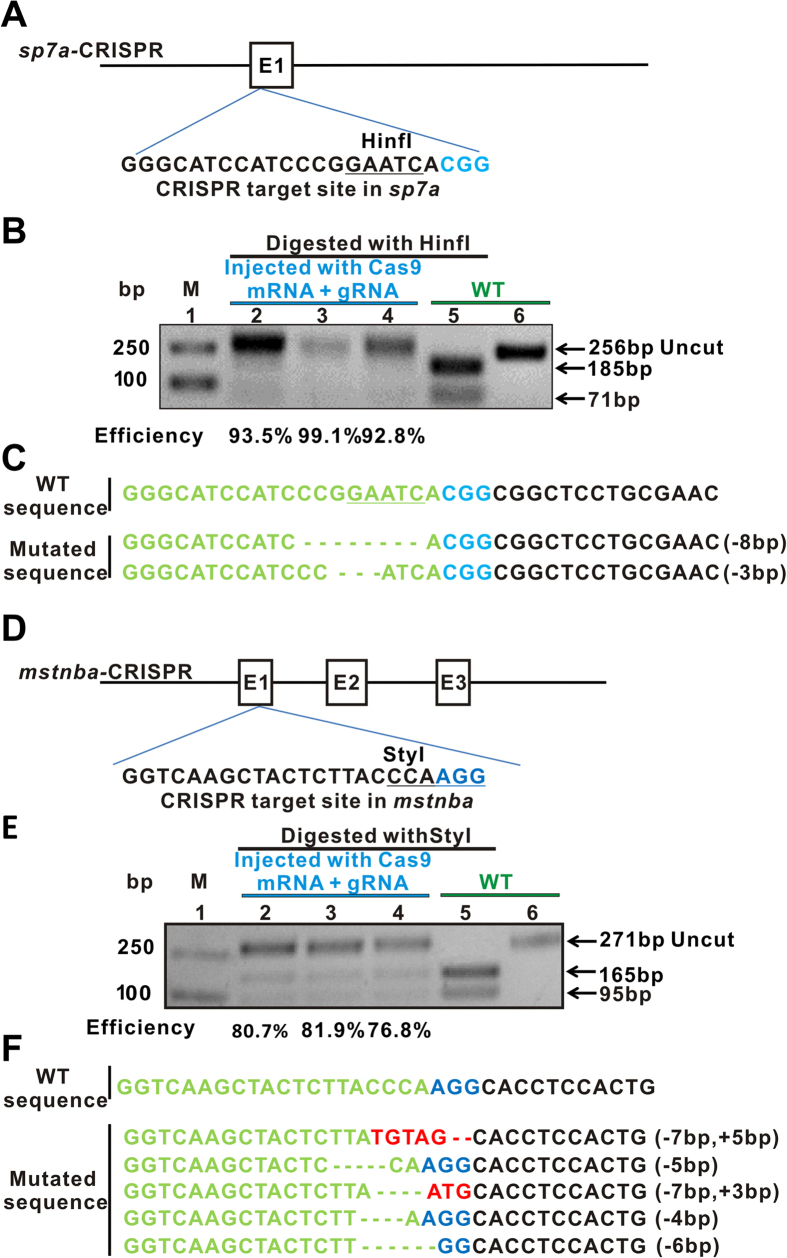
CRISPR-Cas9-induced mutagenesis efficiencies of common carp genes evaluated in common carp embryos. (**A**) Schematic of gRNA-Cas9 targeting the exon of common carp *sp7a*. The restriction enzyme HinfI site in the targeted fragment is underlined. The PAM sequence is highlighted in blue. E: exon. (**B**) Enzymatic digestion analysis of Cas9-mediated cleavage at *sp7a* in carp embryos digested with HinfI. The lane 1, marker; lanes 2–4, injected groups at concentrations of 100 pg, 150 pg and 200 pg of *sp7a g*RNA (below the blue line), respectively; lane 5, wild-type control with digestion (below the green line); lane 6, undigested wild-type control. Uncut (256 bp) and cut (185 bp and 96 bp) bands are indicated. WT, wild type; M, marker. Mutation efficiencies are 93.5% for lane 2, 99.1% for lane 3 and 92.8% for lane 4. (**C**) Indels in the CRISPR-Cas9 *sp7a* targeted site, revealed by sequencing analysis. The gRNA-targeting sequence is labeled in green. (**D**) Diagram of the Cas9-gRNA-targeted site in common carp *mstnba*. The restriction enzyme StyI site is underlined. E: exons. (**E**) Enzymatic digestion analysis of Cas9-mediated cleavage at *mstnba* in carp embryos. The *mstnba*-targeted fragment was PCR amplified and digested with StyI. Lane 1, marker; lanes 2–4 (below the blue line), injected groups at concentrations of 25, 50, 100 pg of *mstnba g*RNA, respectively; lane 5, wild-type control with digestion (below the green line); lane 6, wild-type control without digestion. Uncut (271 bp) and cut (165 bp and 96 bp) bands are indicated. WT, wild type; M, marker. Mutation efficiencies are 80.7% for lane 2, 81.9% for lane 3 and 76.8% for lane 4. (**F**) Indels in the CRISPR-Cas9-targeted *mstnba* site revealed by sequencing. Insertions are labeled in red. Electrophoretic results were cropped from the original images shown in Supplementary Fig. S13F,G.

To examine the CRISPR-Cas9-induced mutagenesis efficiencies, Cas9 mRNAs plus the six individual gRNAs for corresponding genes *sp7a*, *sp7b*, *mstnba*, *runx2*, *opga* and *bmp2ab* were co-microinjected into one-cell common carp embryos, respectively (Fig. 2A). For *sp7a-*CRISPR, enzymatic digestion and sequencing analyses showed that 18 out of the 20 clones tested harbor mutations (Fig. 4A–C), which were reconfirmed with T7E1 enzyme analysis (Supplementary Table S5). For *mstnba-*CRISPR, the DNA fragment containing the targeted site was PCR amplified from 72 hpf embryos, and digested with the Styl enzyme. The results showed that the mutation efficiency was more than 70% (Fig. 4D,E), consistent with T7E1 enzymatic digestion and DNA sequencing analyses (Fig. 4F and Supplementary Table S5). In addition, *runx2*-, *sp7b*- and *opga*-CRISPR-Cas9 also produced gene mutations with efficient efficiencies more than 50%, but *bmp2ab*-CRISPR had relatively low mutation efficiency of approximately 40% in carp embryos (Supplementary Figs S4–S7). The results of all targeted genes of common carp in F_0_ generation are shown in Supplementary Table S5.

### More muscular cells in *mstnba* mutant common carps

**Figure 5:**
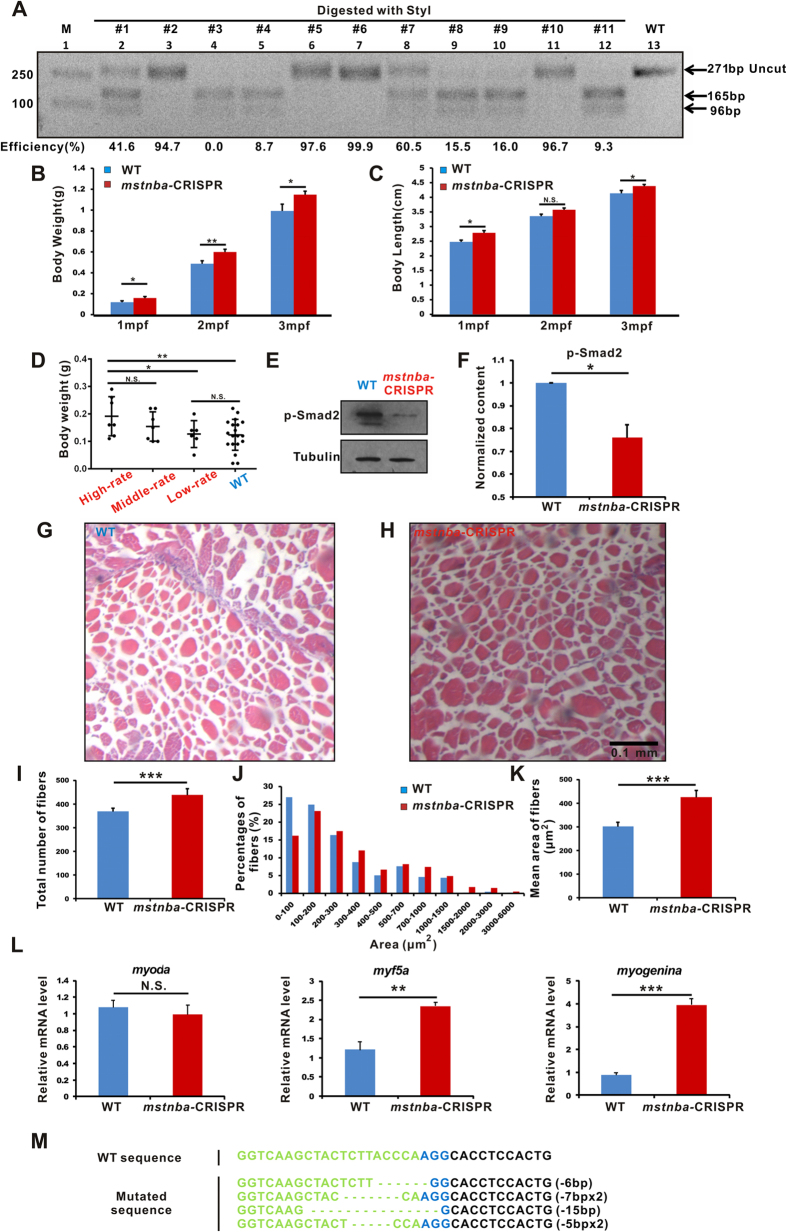
More muscular cells in CRISPR-Cas9-targeted *mstnba* common carps. (**A**) Enzymatic digestion of CRISPR-Cas9-mediated cleavage at *mstnba*. PCR products were digested with StyI. Lane 1, marker; lanes 2–12, digestion of PCR products of 11 carps with StyI; lane 13, wild-type without digestion. Uncut (271 bp) and cut (165 bp and 95 bp) bands are indicated. Each “#” represents a single fish, and estimated mutagenesis efficiencies are indicated at the bottom. Body weight (**B**) and body length (**C**) of one-month-old, two-month-old and three-month-old *mstnba*-CRISPR mutant (red) and wild-type (blue) carps. Values are means ± SEM (n = 20). (**D**) Comparisons of body weights in different genotypes of *mstnba*-CRISPR F_0_ at 1 mpf. High-rate represents higher than 90% of mutation efficiencies in (**A**) middle-rate 10–90%, and low-rate 0–10%. Each black dot represents one sample. Values are means ± SD (n = 6 or 7). (**E**) Western blotting analysis of phosphorylated Smad2. Proteins were obtained from dorsal muscles of *mstnba*-CRISPR (2#, 6#, 10#) and wild-type (3 samples) carps at 5 mpf. (**F**) Quantification of Western blotting results with ImageJ. Values are means ± SD. n = 3. (**G**,**H**) Representative images of muscle cells of three-month-old wild-type (**G**) and 7# *mstnba* mutant carps (**H**), shown by H&E staining. Scale bar: 0.1 mm. Numbers of muscle fibers (**I**) and the average area of the muscle fibers (**K**) of *mstnba* mutant and wild-type carps, quantified H&E staining images (see **G**,**H**) with ImageJ. Approximately 24 stained images each were quantified. (**J**) Distribution analysis of the fiber area. Values are means ± SEM. (**L**) Quantitative RT-PCR analysis of *myoda*, *myf5a* and *myogenina* in *mstnba*-CRISPR (red) and wild-type (blue) carps. RNAs were extracted from dorsal muscles of wild-type (3 samples) and *mstnba*-CRISPR carps (2#, 6#, 10#) at 5 mpf. Values are means ± SD. n = 3. (**M**) Mutations in *mstnba* CRISPR-Cas9 7# carp, revealed by sequencing analysis. The targeted sequence is in green, and the PAM sequences are in blue. Electrophoretic and Western blotting results were cropped from the original images shown in Supplementary Fig. S13I,L. Two-tailed Student’s *t*-test or one-way ANOVA with LSD were conducted, *P < 0.05, **P < 0.01 ***, *P* < 0.001, and N.S., no significant change.

To generate *sp7a*-CRISPR and *mstnba*-CRISPR targeted common carp fish, more than 200 embryos were microinjected for each group. Most of the microinjected and uninjected control fish were put into the ponds when they were 30 days old. In addition, 20 carps each for wild type, *sp7a*-CRISPR and *mstnba*-CRISPR were raised separately in the laboratory under the same condition. All the phenotypes were examined from these 20 carps for each group. PCR products amplified with DNAs extracted from caudal fins of one-month-old common carps were digested with restriction enzyme StyI for *mstnba-*CRISPR. Results showed *mstnba*-CRISPR had high mutation efficiency in the somatic cells of one-month-old carp, averaging at 56.6% for the 20 carps, similar to the sequencing results in carp embryos (Fig. 5A).

The body weight and body length of these microinjected (all the 20 carps in the group including 3# in Fig. 5A with no detectable mutations) and uninjected control common carps were measured at one mpf (month postfertilization), two mpf and three mpf. Unsurprisingly, *mstnba*-CRISPR common carps grew heavier than uninjected controls in all three time points and longer than uninjected controls in 1 mpf and 3 mpf (Fig. 5B,C). Body weights (Fig. 5B) were significantly increased at 1 mpf (*P* < 0.03), 2 mpf (*P* = 0.005) and 3 mpf (*P* < 0.049) in *mstnba*-CRISPR carp compared with wild types. Body lengths (Fig. 5C) were also significantly increased in *mstnba*-CRISPR carp compared with wild types at 1 mpf (*P* = 0.011) and 3 mpf (*P* = 0.047). We selected 7 *mstnba*-CRISPR carps with more than 90% mutation efficiency as the high-rate subgroup, 7 *mstnba*-CRISPR carps with 10–90% mutated efficiencies as the middle-rate subgroup and 6 *mstnba*-CRISPR carps with less than 10% mutation efficiency as the low-rate subgroup. Body weights were significantly increased (*P* = 0.007) in high-rate subgroup compared with wild types, and in high-rate subgroup (*P* = 0.049) compared with low-rate subgroup, implicating that the body weight exhibits a phenotype-genotype correlation, *i.e.*, the higher the mutation rate (the high-rate subgroup), the heavier the fish compared with the low-rate subgroup (Fig. 5D). We also examined the level of phosphorylated Smad2, which is an essential intracellular transducer for the TGF-β signaling pathway^51^, in dorsal muscles of five-month-old *mstnba*-CRISPR and wild-type common carps with high mutation rate by Western blotting. Results showed approximately 25% reduced phosphorylated Smad2 in *mstnba*-CRISPR common carps (*P* = 0.024). (Fig. 5E,F), implicating that CRISPR-Cas9-induced mutations impair the Mstn signal pathway of the skeletal muscle of common carp. Moreover, H&E (hematoxylin and eosin) staining of the dorsal muscle of 7# carp in Fig. 5A showed significantly higher numbers of muscle cells and muscle fibers, and significantly increased size of muscle fibers in *mstnba*-CRISPR carp displays in comparison with uninjected controls (Fig. 5G–K). We also analyzed dorsal muscles of five-month-old *mstnba*-CRISPR with high mutation rate and wild-type common carps using qRT-PCR. Results showed significant up-regulation of myogenic regulatory factors *myf5a* (a hyperplasia marker)^51^ (*P* < 0.01) and *myogenina* (a hypertrophy marker)^51^ (*P* < 0.001) but no difference for *myoda* (a hyperplasia marker)^51^ (*P* = 0.353) in mutant carps (Fig. 5L). Together, these results indicate that *mstnba*-CRISPR mutated F_0_ carps showed hyperplasia as well as hypertrophy^51^. Sequencing analysis of 7# carp in Fig. 5A showed that six out of the 10 single clones were mutated, and most of the mutation types are frameshifters resulting in truncated peptides, even though mutations are likely mosaic (Fig. 5M). The phenotypes of increasing the body size and muscles of *mstnba*-CRISPR carp are consistent with those of the *Mstn*-mutated animals^45^.

### Severe bone defects in *sp7a* mutant common carps

**Figure 6:**
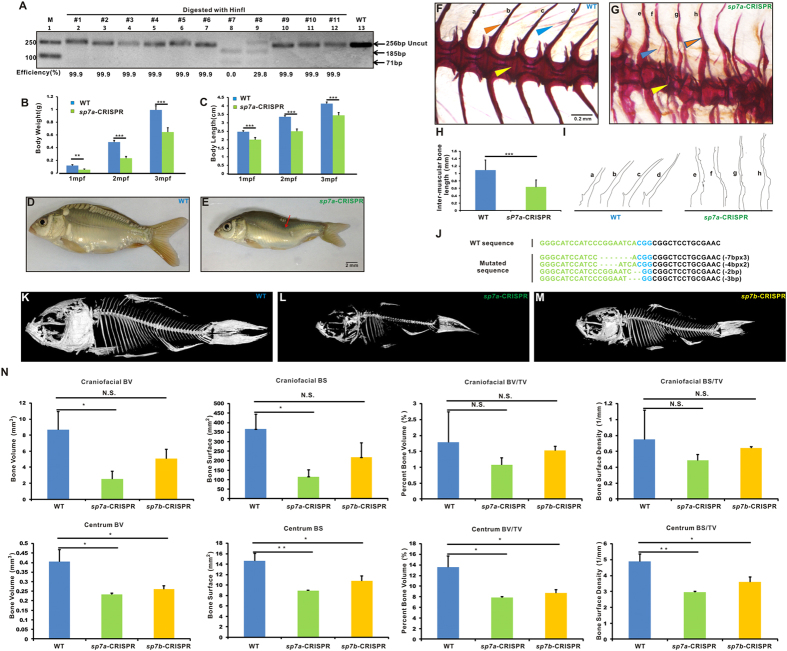
Severe bone defects in CRISPR-Cas9-targeted *sp7a* common carps. (**A**) Enzymatic digestion of CRISPR-Cas9-mediated cleavage at common carp *sp7a*. PCR products were digested with HinfI. Lane 1, marker; lanes 2–12, digestion of PCR products form 11 injected carps with HinfI; lane 13, wild-type without digestion. Uncut (256 bp) and cut (185 bp and 71 bp) bands are indicated. Electrophoretic results were cropped from the original images shown in Supplementary Fig. S13H. Body weights (**B**) and lengths (**C**) were significantly decreased at 1 mpf (*P* < 0.01), 2 mpf (*P* < 0.001) and 3 mpf (*P* < 0.001) in *sp7a*-CRISPR carp (green) compared with wild types (blue). Values are means ± SEM. n = 20 per sampling stage. Images of one-month-old wild-type (**D**) and *sp7a* CRISPR-Cas9-induced mutant 3# (**E**) carps. Red arrow indicates the deformed bones. Scale bar, 2 mm. Alizarin Red staining of vertebra bones for one-month-old wild-type (**F**) and *sp7a*-CRISPR-Cas9 mutant 3# (**G**) carps. Blue arrowheads indicate intermuscular bones, orange arrowheads hemal spine bones and yellow arrowheads the centrums region. Scale bar, 0.2 mm. (**H**) Average length of inter-muscular bones in wild-type (blue, three carps) and *sp7a*-CRISPR (green, eight carps from Supplementary Fig. S8). All the 32 inter-muscular bones in each carp were quantified with ImageJ. Values are means ± SD. (**I**) Silhouettes scaled to the same final magnification to illustrate the shape of hemal spine bones. a-h are different hemal spine bones from wild types (a–d) and *sp7a* CRISPR-Cas9 3# (e–h) in (**F**,**G**). (**J**) Mutations in *sp7a* CRISPR-Cas9 3# carp, revealed by sequencing analysis. The gRNA-targeted sequence is in green, and the PAM sequences are in blue. Micro-CT images of two-month-old wild-type control (**K**), *sp7a* CRISPR-Cas9-induced 11# mutant (**L**) and *sp7b* CRISPR-Cas9-induced mutant (**M**) carps. (**N**) BV (bone volume), BS (bone surface), BV (bone volume)/TV (tissue volume) and BS (bone surface)/TV (tissue volume) of cranioficial bones (up panels) and centrum bones (bottom panels) in *sp7a*-CRISPR (green), *sp7b*-CRISPR (yellow) and WT control (blue), calculated from the Micro-CT analysis. n = 3. Values are means ± SD. Two-tailed Student’s *t*-test or one-way ANOVA with LSD were conducted, *P < 0.05, **P < 0.01, ***P < 0.001 and N.S., no significant difference.

PCR products amplified with DNAs extracted from caudal fins of one-month-old common carps were digested with restriction enzyme HinfI for *sp7a-*CRISPR. Results showed that 16 out of the 18 *sp7a*-CRISPR injected carp were mutated, suggesting that almost all the somatic cells of the fin were mutated in the *sp7a* target site of common carp (Fig. 6A).

In contrast to *mstnba*-CRISPR mutant carps, *sp7a*-CRISPR mutants carps grew lighter and smaller than uninjected wild-type controls in all three time points (*P* < 0.001) (Fig. 6B,C). *sp7a*-CRISPR common carps exhibit conspicuous bone defects including opercula and maxilla insufficiency and bending back such as 3# *sp7a*-CRISPR carp in Fig. 6A (Fig. 6D,E). Alizarin Red staining of 3# *sp7a*-CRISPR carp showed that the irregular hemal spines and deformed centrums, and shorter inter-muscular bones than uninjected wild-type control (Fig. 6F–I). Sequencing analysis of the fin-clipped DNA of 3# *sp7a*-CRISPR carp showed that all the seven single clones were mutated (Fig. 6J). We selected eight carps including 1# to 06#, 9# and 10# in Fig. 6A, which all showed nearly 100% mutated rate, for Alizarin Red staining. Results showed four out of the eight carps display the crinkled neural spines and deformed centrums, and three out of the eight fishes display curved hemal spines and maxilla insufficiency (Supplementary Figs S8 and S9). Micro-CT analysis showed that although both *sp7a*-CRISPR (11#, 12# and 13#, in Fig. 6L is 11#) and *sp7b*-CRISPR (1#, 2#, 3#, in Fig. 6M is 1#) showed smaller bone size compared with control group, *sp7a*-CRISPR carps showed more obvious bone defects than *sp7b*-CRISPR. The craniofacial bones and centrum bones of these *sp7* mutated carps develop slowly than those of control groups (Fig. 6K–M). Further, the bone volume (BV), bone surface (BS), bone volume (BV)/tissue volume (TV) and bone surface (BS)/tissue volume (TV) in centrum bones are statistically significantly reduced in *sp7a*-CRISPR (*P* < 0.05) and *sp7b*-CRISPR (*P* < 0.05) compared with wild types (Fig. 6N). The bone volume (BV) and bone surface (BS) of craniofacial bones of *sp7a*-CRISPR carps are also significantly reduced (*P* < 0.05). However, neither the bone volume (BV)/tissue volume (TV) and bone surface (BS)/tissue volume (TV) of craniofacial bones of *sp7a*-CRISPR carps (*P* > 0.2), nor the bone volume (BV), bone surface (BS), bone volume (BV)/tissue volume (TV) and bone surface (BS)/tissue volume (TV) in craniofacial bones of *sp7b*-CRISPR carps are significantly reduced (*P* > 0.1) (Fig. 6N). In addition, *sp7a*-CRISPR carps display small-sized and irregular-shaped scales and fewer pharyngeal teeth compared with wild types (Supplementary Figs S10 and S11). Together, these results indicate highly efficient CRISPR-Cas9 may generate bialleic mutations resulting in obvious phenotypes in F_0_-generation common carp.

It usually takes 3 to 4 years for common carp to grow sexually mature. To examine the possibility of germline transmission, DNAs were extracted from the testis of 5# *mstnba*-CRISPR carp that has nearly 100% mutation rate based upon analysis of fin-clipped DNAs. Sequencing data show 10 out of the 10 single clones (100%) were mutated (Supplementary Fig. S12), whereby implicating that these mutations are likely transmitted to the subsequent generation.

### Multiplex gene-editing in a single common carp

**Figure 7:**
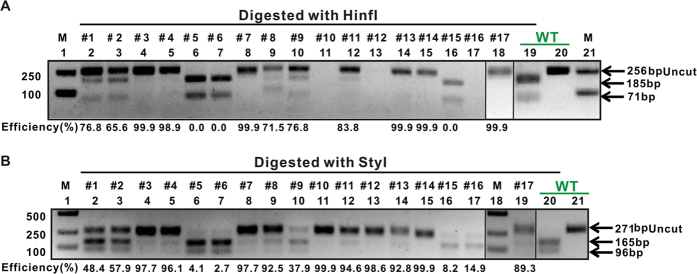
Modification of double genes *sp7a* and *mstnba* in a single common carp. (**A**) Enzymatic digestion analysis of one-month-old *sp7a;mstnba*-Cas9 double-mutated common carps with HinfI. Digestion of PCR products from 17 one-month-old *sp7a;mstnba*-Cas9 double-injected carps with HinfI for *sp7a* (below the black line). Estimated mutagenesis efficiencies are indicated at the bottom. Lanes 1, 21, markers; lanes 2–18, digestion of PCR products amplified using fin-clipped DNAs of 17 one-month-old carps with HinfI; lane 19, wild-type control with digestion (below the green line); lane 20, wild-type control without digestion. Uncut (256 bp) and cut (185 bp and 71 bp) bands are indicated. (**B**) Enzymatic digestion analysis of one-month-old *sp7a;mstnba*-Cas9 double-mutated carps with StyI. Digestion of PCR products from the same 17 one-month-old *sp7a;mstnba*-Cas9 double-injected carps with StyI for *mstnba* (below the black line). Estimated mutagenesis efficiencies are indicated at the bottom. Lanes 1 and 18, markers; lanes 2–17, 19, digestion of PCR products amplified using fin-clipped DNAs of the same17 one-month-old carps with StyI; lane 20, wild-type control with digestion (below the green line); lane 21, wild-type control without digestion. Uncut (271 bp) and cut (165 bp and 96 bp) bands are indicated. Electrophoretic results were cropped from the original images shown in Supplementary Fig. S13J,K.

Common carp is a tetraploidy species with 100 chromosomes and the high DNA content^2^. The ability to modify duplicated genes or multiple genes of common carp is important for its molecular genetic studies and breeding. To address this issue, *sp7a*-CRISPR and *mstnba*-CRISPR were co-microinjected into common carp embryos. PCR products amplified with *sp7a* and *mstnba* primers and DNAs extracted from caudal fins of one-month-old injected and uninjected control carps were digested with HinfI or StyI, respectively. The results showed that 16 out of the 21 carps had both gene mutations. Mutation efficiencies was 63.4% for *sp7a* gene, and 60.1% for *mstnba* gene, and among them, eight carps had more than 90% efficiencies in these two genes (Fig. 7A,B). These results indicated that double-gene-editing are feasible in common carp with CRISPR-Cas9 in a single step.

## Discussion

Due to the tetraploidy genome of common carp, it is extremely difficult to obtain the homozygous mutants using the traditional methods such as ENU, TILLING, and retroviral insertion. However, using TALEN and CRISPR-Cas9 methods we have successfully targeted carp genes of interest and observed the specific phenotypes in F_0_ generation. We used TALEN to target four genes, *sp7a*, *runx2*, *mstnba* and *spp1a* and CRISPR-Cas9 to target six genes, *sp7a*, *sp7b*, *runx2*, *mstnba*, *opga* and *bmp2ab* with high efficiencies. With TALEN, we have achieved higher mutagenesis efficiencies with average of 31.83% in common carp than previously reported 3.0% to 12.4% in zebrafish^12^. We also observed that the shorter the spacer length results in the higher mutagenesis in common carp with TALEN, consistent with previous studies^49^. The highest efficiency of common carp TALEN is *spp1a*-TALEN that has the shortest spacer length of 15 bp.

Even though TALEN and CRISPR-targeted F_0_ fish were genotypically complex, the highly induced mutagenesis frequencies of somatic mutations still can result in obvious phenotypes in F_0_ founders, as reported in mice and zebrafish^24^,^33^. Hence CRISPR-Cas9 can have the similar effect like Morpholinos^33^ to alter the gene functions in common carp. It is applicable to the genes that function in later development or in adulthood such as mature osteoblast genes, circadian clock genes, and ageing genes.

Although *sp7a*-targeted site with CRISPR-Cas9 has a nearly 100% mutation efficiency in embryos and somatic cells, its mutation types are different among the different cells (Fig. 6J). CRISPR-Cas9-induced mutations are complex in F_0_ founder carps. First, these mutations could be mosaic, meaning that the same mutations may be located in different tissues or organs^33^,^52^, and in some cases the targeted tissues or organs may not carry the mutations and thus display no expected phenotypes^33^,^52^. Second, several different types of mutations could be in different tissues or organs, for example, the mutated sequences of *mstnba*, some causing truncated peptides and the other resulting in only deletion or insertion of a few amino acids, are different among different types of cells, as shown in Fig. 5M and Supplementary Fig. S12. Therefore the phenotypes of the body weight vary in the same group of carps (Fig. 5D). The complex nature of CRISPR-Cas9-induced mutations is also responsible for variations of Alizarin Red staining of *sp7a*-CRISPR F_0_ carps (Supplementary Figs S8 and S9) and only 25% reduced levels of phosphorylated Smad2 (Fig. 5E,F). Another aspect of the complexity of phenotypes observed in F_0_ founder carps is that there are multiple copies of genes under study, for instance, four *mstn* genes in common carp, and usually only one gene is mutated (Fig. 1B). A possible way to obtain a consistent phenotype in the F_0_ founder carp is to target the important functional domains such as the Z-F (Zinc-Finger) motif of *sp7* with high mutation rate, which would induce biallelic mutations.

Sp7/Osterix plays an essential role in differentiation and maturation of osteoblasts and formation of osteocytes but not in cartilage development in mice and medaka^35^,^53^. We observed that body weight is lighter in *sp7a*-CRISPR mutated F_0_ carps than wild-type control, maybe because the delayed and abnormal osteoblast development; some mutant carps have curved spines, the other mutant carps have abnormal craniofacial bones (Fig. 6D–I and Supplementary Figs S8 and S9). Further, micro-CT analysis showed the bone volume (BV), bone surface (BS), bone volume/tissue volume (BV/TV) and bone surface/tissue volume (BS/TV) are significantly reduced in *sp7a*-CRISPR carps (Fig. 6N). Together, these results strongly indicate that the observed phenotypes were resulted from the disruption of *sp7a* gene, consistent with previous studies^35^,^53^,^54^. In contrast to the mouse model died after birth^35^, *sp7* mutant carps can survive at least 3 months, providing a unique opportunity for investigating roles of Sp7 in bone development. In particular, the inter-muscular bones are shorter in of *sp7a*-CRISPR carps in comparison with wild types (Fig. 6H) but their number are not changed, demonstrating that Sp7a plays a role in common carp inter-muscular bone development. Even though *sp7a*-CRISPR mutated carps are not good aquaculture lines due to the abnormal bones and lighter body weight, they are certainly useful for finding carps without or with fewer inter-muscular bones and investigating teleost osteoblast formation.

We have used the CRISPR-Cas9 method to generate mutant carps for multiple genes with high efficiencies. Many phenotypes are derived from mutations in multiple genes rather than a single gene. There are a lot of genes with multiple copies in common carp due to additional round of genome dupolication^55^. Hence mutagenizing double genes or multiple genes with high efficiencies would facilitate generation of multiple-gene-mutated carps. In addition, we can mutagenize multiple copies simultaneously in a single carp and study their functions. Future work will use single gRNA to modify multiple homologous genes as described previously in *Xenopus Laevis*^56^.

For a large aquatic species such as common carp, it is important to generate mutants with high germline transmission frequencies to reduce the cultivation time, pool space and labor force. Many studies have been focused on increasing the targeting efficiency^18^,^20^. In this study, we have achieved mutagenesis efficiencies up to 75% for TALEN and up to 100% for CRISPR-Cas9, not only in embryos and juveniles examined, but also in the testis. Our examination of the testis indicated that almost all the testis cells including spermatogonia or spermatocytes are biallelically mutated, implicating that these CRISPR-Cas9-induced mutations are likely transmitted to the subsequent generation. A study of the F_0_ monkey placenta showed that the germline cells likely would be modified by CRISPR-Cas9^57^. Zebrafish studies showed that 87.3% mutated efficiency for *gol* in F_0_ somatic cells^33^ and 52.7% mutated efficiency for *fh* in F_0_ somatic cells^58^ with CRISPR-Cas9 both led to 100% germline transmission rate. Another zebrafish study showed that 12.5% mutated rate for *tnikb* in F_0_ somatic cells with TALEN led to 33.3% germline transmission rate^12^. Similar to these previous studies, we should be able to obtain germline-transmitted carp lines with as high as 100% of somatic mutation rate in TALEN- and CRISPR-Cas9-targeted genes.

In summary, our studies demonstrate that TALEN and CRISPR-Cas9 technologies are effective tools for genetics studies in common carp, which will greatly promote genetic engineering in aquaculture and have the potentials for improving their qualities and economic value in the future. To our knowledge, this is the first report on targeted disruption of endogenous genes in common carp using TALEN and CRISPR-Cas9.

## Methods

All animal care and experiments were performed in accordance with the institutional ethical guidelines for animal experiments, and all fish experimental procedures were approved by the Soochow University Committee on Animal Use and Care.

### Design of TALEN and CRISPR target sites

The TALEN sites were selected by “TAL Effector Nucleotide Targeter 2.0” to target on the first exon of most genes^59^. An enzyme site in the spacer was selected if it is available for genotyping. Blasting the target sequences against the whole genome DNA database (http://www.carpbase.org/login.phpweb address) of common carp was performed to avoid the off-target sites.

The gRNAs for CRISPR-Cas9 were designed by “seqbuilder” software according to the 5′-GGNNNNNNNNNNNNNNNNNNNGG-3′ roles^13^. The first two G are necessary for the T7 RNA polymerase and the end NGG is the PAM. The minmal number of nucleotides (N) is 19 bp depending on the sequence for each gene. A restrictive enzyme near the PAM was also selected if it is available.

### Construction of TALEN and synthesis of Cas9 and gRNAs

The two arms of TALEN were constructed using the “Unit Assembly” method^12^. Simply, customized TALE repeats were inserted into the 5′-end of the backbone vector (PMD-19T) by double-digesting with either NheI and HindIII or SpeI and HindIII. The final pMD-TALE repeats were confirmed by sequencing with the M13 primer. Then, the DNA fragments digested with NheI and SpeI, were ligated into the TALEN expression vectors-PCS2-PEAS and PCS2-PERR. The final constructs were linearized with NotI and used as templates for TALEN mRNA synthesis with SP6 mMESSAGE mMACHINE Kit (Ambion).

The Cas9 mRNA and gRNAs were synthesized as described previously^29^,^33^ with modifications. Briefly, the Cas9 mRNA was synthesized by *in vitro* transcription using T7 mMESSAGE mMACHINE Kit (Ambion). The DNA templates of gRNA were generated by PCR with a pair of primers (Supplementary Table S6), and then purified by phenol and chloroform. gRNAs were *in vitro* transcribed with SP6 Riboprobe Systems (Promega), and purified with lithium chloride and ethanol precipitation.

### Zebrafish maintenance and microinjection

Zebrafish (*Danio rerio*) are maintained at 28.5 °C under 14 h light:10 h dark (14:10 h LD) cycles at the Soochow University Zebrafish Research Facility. To test the targeting efficiency of TALEN pairs and the CRISPR-Cas9 system, 50–100 pg purified plasmid containing gene specific DNA fragment of carp plus 250 pg TALEN mRNAs of each arm or 300 pg Cas9 mRNA plus 25–100 pg carp gRNAs were microinjected into one cell-stage zebrafish embryos. Genomic DNAs extracted from injected embryos at 24 hpf (hours postfertilization) were used as templates for PCR.

### Artificial insemination of common carp

The brood stocks of common carp were obtained from The Heilongjiang Fisheries Research Center, Harbin, Heilongjiang, China, and are maintained at the New Era Fish Breeding Facility of Xiangcheng, Suzhou, Jiangsu, China. Well-developed 3-year-old brood fish with the average body weight of 2 kg of adult carp were selected. Female fish were injected with 5 ug/kg luteinizing hormone-releasing hormone A2 (LHRH-A2, Ningbo secondary Hormone Company, China) and 1 mg/kg domperidone (DOM, Ningbo secondary Hormone Company, China) together dissolved in 0.68% physiological saline in a total volume of 2 ml, while male fish received only half of this dosage. Male and female fishes were kept separately in circular breeding ponds with flowing water stimulation. Then common carp eggs were manually stripped into a dry centrifuge tube, while milts were manually stripped from the males into a centrifuge tube with 10 ml Hank’s solution (0.4 g KCl, 8 g NaCl, 0.35 g NaHCO3, 0.09 g NaH2PO4.7H2O, 0.1 g MgSO4.7H2O, 0.1 g MgCl2.6H2O, 0.06 g KH2PO4, 0.14 g CaCl2 and 1 g glucose in 1 L sterile deionized water) for *in vitro* fertilization.

### Microinjection and maintenance for the embryos of common carp

Microinjection began at 20 min after fertilization. Short but sharp needles were pulled by the Micropipette Puller (P-1000, Sutter Instrument) using the thin wall borosilicate tubing with filament (Catalog Number: BF120-94-10; Outside Diameter: 1.20 mm; Inside Diameter: 0.94 mm; Overall Length: 10 cm; Sutter Instrument), and the program of pulling the needle was Heat: 750; Pull: 80; Vel: 55; Time: 250; Pressure: 500; and Ramp: 765.

For the injection of the TALEN arms, 250 pg TALEN mRNAs of each arm were co-microinjected into one-cell carp embryos; For CRISPR-Cas9, 300 pg Cas9 mRNA and 25–200 pg gRNA were co-injected into the embryos. Microinjected embryos were grown at room temperature and collected for DNA extraction at 48–72 hpf.

### Common carp larval feeding and maintenance

Four dpf-12 dpf larvae were fed with paramecia; then fed with brine shrimps until 25 dpf. 20–90 dpf juveniles were fed with the eel powder for two days, and then were transferred into pools. Those fish to be examined were taken to the laboratory. Each fish was raised in a 3-L tank at 22 °C under 14 h light:10 h dark (14:10 h LD) cycles and fed with brine shrimp and eel powder. The food was fed equivalently in all the groups.

### Mutagenesis efficiency analyses in injected embryos

#### Restriction digestion assay

TALEN- and CRISPR-Cas9/gRNA-induced mutation efficiencies were examined by the restriction digestion assay as described previously^18^. PCR products were digested with corresponding restriction endonuclease enzymes for 2 h at 37 °C. The intensities of cleaved and uncleaved bands were quantified with the ImageJ software.

#### T7 endonuclease I (T7EI) assay

300 ng of purified PCR products were denatured and slowly re-annealed to facilitate heteroduplex formation. The re-annealing procedure includes a 5-min denaturing step at 95 °C, followed by cooling to 85 °C at −2 °C/sec and further to 25 °C at −0.1 °C/sec. The re-annealed amplicon was then digested with 10 U of T7E1 endonuclease I (New England Biolabs) at 37 °C for 90 min, and digested amplicon was electrophoresed on a 2% agarose gel. Band intensities were quantified with the ImageJ software.

#### DNA sequencing

For DNA sequencing, DNA fragments containing the targeted sites were PCR amplified with DNAs extracted from caudal fins of 1-month old carps, and cloned into the PMD-19T vector. Single clone was picked up, verified by PCR and sequenced by Sanger sequencing (GENEWIZ, Inc.).

### RNA isolation, RT-PCR and quantitative real-time PCR analyses

Total RNAs were extracted from the maw, liver, gill, eye, heart, brain, gut, testis and muscle of 3-month-old common carp fishes with TriZol (Invitrogen, CA, USA), and reverse transcribed into cDNAs with Superscript III Reverse Transcriptase (Invitrogen, CA, USA), respectively. RT-PCRs were carried out with the following thermal profile of 94 °C for 5 min and 94 °C, 20 sec; 58 or 60 °C, 20 sec, 72 °C, 15 sec for 38 cycles. Quantitative real-time PCR (qRT-PCR) was performed in an ABI StepOnePlus instrument with the SYBR green detection system (Invitrogen) using a PCR thermal profile with 40 cycles of 10 s at 95 °C and 30 s at 60 °C. All results were normalized to the expression level of the housekeeping gene β-actin. qRT-PCR results are shown as a relative expression level calculated using the 2 ^−△△CT^ method^60^. Primers for *mstnaa, mstnab, mstnba*, *mstnbb, myoda, myf5a* and *myogenina* are listed in Supplementary Table S6. Each PCR assay was done with three biological samples. *P* values were calculated with Student’s *t* test.

### Alizarin red staining

Mineralization of calcium deposits was assessed by Alizarin Red S (Sigma) staining conducted as previously described^61^. Three-month-old carps from mutant and control groups were anesthetized with 0.03% Tricaine (Sigma, USA). The stained matrix was examined using a Leica stereomicroscope and was photographed using an Olympus 35-mm camera (Olympus, Tokyo, Japan).

### H&E (Hematoxylin and eosin) staining

Three-month-old carps from mutant and control groups were anesthetized with 0.03% Tricaine (Sigma, USA). Subsequently, carp dorsal muscles were dissected out and fixed in 4% paraformaldehyde at room temperature for 24 hours, and then sectioned and stained with hematoxylin/eosin as described previously^51^,^62^. Dorsal muscles of carp pelvic fins were counted. Cell number was calculated as the number of fibers per cross-sectional muscle area. The muscle fiber numbers and area were determined with the ImageJ program. The muscle numbers were calculated by the “Cell Counter” features of ImageJ.

### Micro computed tomography (Micro-CT)

Two-month-old common carp with approximately 3.5 cm of body length were anesthetized with 0.03% Tricaine (Sigma, USA) and gently put into 5 ml centrifuge tubes. The whole body was imaged in a SkyScan 1176 high-resolution micro-CT scanner (Skyscan, Kontich, Belgium) using a 8.8 μm voxel size. The applied X-ray voltage was 45 kV and current was 550 uA. Scans were over 180° with a 0.7° rotation step. Images were reconstructed and binarised with global thresholding using SkyScan CT Analyser software (Version: 1.10.11.0), as described^63^. A region of interest was traced around individual whole body or head models prepared using the “Double Time Cubes” 3D reconstruction method. Cortical bone mineral density (BMD) was estimated by comparing bone density with calibration phantoms of known BMD, scanned at the same time as the head.

### Western blotting analysis

Proteins extracted from dorsal muscles of five-month-old common carps were washed with fish water and homogenized in lysis buffer as described previously^60^. Protein samples were separated with sodium dodecyl sulfate-poly-acrylamide gel electrophoresis (SDS-PAGE) using 5% acrylamide stacking gel followed by 10% gradient separation gel and transferred to NC membranes. After blocking with 5% nonfat milk (blocking solution) for 1 h at room temperature. The blocking solution was also used as dilution solutions for antibodies. NC membranes were incubated with primary antibody overnight at 4 °C; anti-phospho Smad2 (1:500, Millipore Corp., MA, USA), anti-Tubulin (1:1000, Cell Signaling Technology, Inc., MA, USA). The next day, membranes were washed in TBST (0.5% Tween 20) four times, each for 5 min, and then incubated for 2 h with goat anti-rabbit HRP-conjugated secondary antibody (1:10000; Santa Cruz Biotechnology, Inc., CA, USA) at room temperature. After washing with TBST four times, 10 min each, membranes were detected by Amersham ECL prime (GE Healthcare). Equal loading of the proteins was confirmed with an anti-Tubulin antibody.

### Statistical analysis

Statistical analyses were performed with the unpaired, two-tailed Student’s *t*-test or one-way ANOVA with *post hoc* LSD method. All statistical analyses were executed with SPSS 16.0 software and *P* < 0.05 was regarded as a statistically significant difference, *P < 0.05, **P < 0.01, ***P < 0.001.

## Additional Information

**How to cite this article**: Zhong, Z. *et al.* Targeted disruption of *sp7* and *myostatin* with CRISPR-Cas9 results in severe bone defects and more muscular cells in common carp. *Sci. Rep.* **6**, 22953; doi: 10.1038/srep22953 (2016).

## Supplementary Material

Supplementary Information
