# Supplementary material for: Targeted disruption of sp7 and myostatin with CRISPR-Cas9 results in severe bone defects and more muscular cells in common carp
Source: Sci Rep. 2016 Mar 15;6:22953. doi: 10.1038/srep22953 (PMC4791634; doi:10.1038/srep22953)
Supplement: Supplementary Information [file srep22953-s1.pdf]

Supplementary Information

Targeted disruption of *sp7* and *myostatin* with CRISPR-Cas9 results in severe bone defects and more muscular cells in common carp

Zhaomin Zhong [1,2,*], Pengfei Niu [1,2,*], Mingyong Wang [1,2], Guodong Huang [1,2], Shuhao Xu [1,2], Yi Sun [1,2], Xiaona Xu [1,2], Yi Hou [2], Xiaowen Sun [3], Yilin Yan [1,2,4], Han Wang [1,2,§]

[1]Center for Circadian Clocks, Soochow University, Suzhou 215123, Jiangsu, China

[2]School of Biology & Basic Medical Sciences, Medical College, Soochow University, Suzhou 215123, Jiangsu, China

[3]Heilongjiang River Fisheries Research Institute of Chinese Academy of Fishery Sciences, Harbin, China

[4]Institute of Neuroscience, University of Oregon, Eugene, Oregon 97403, USA

*These authors contributed equally to this work.

[§]Corresponding author: Han Wang, Center for Circadian Clocks, Soochow University, 199 Renai Road, Suzhou, Jiangsu 215123, China. Tel: +86 51265882115; Fax: +86 51265882115; han.wang88@gmail.com or wanghan@suda.edu.cn.

Six supplementary Tables S1-S6 and 13 supplementary figures S1-S13 are included as follows:

Supplementary Table S1 Toxicities of TALEN mRNAs and gRNA-Cas9 nucleases in zebrafish embryos.

Supplementary Table S2 TALEN- or CRISPR-Cas9-induced mutagenesis efficiencies evaluated in zebrafish embryos.

Supplementary Table S3 Mutation rates of each gene using the TALEN system in common carp embryos at 48-72 hpf.

Supplementary Table S4 Comparisons of mutation rates using the TALEN system based upon the spacer length.

Supplementary Table S5 Mutation rates of each gene using the CRISPR-Cas9 system in common carp embryos at 48-72 hpf.

Supplementary Table S6 PCR primers.

Supplementary Figure S1 Evaluation of TALEN-induced mutagenesis efficiencies for *runx2* in common carp embryos.

Supplementary Figure S2 Evaluation of TALEN-induced mutagenesis efficiencies for *mstnba* in common carp embryos.

Supplementary Figure S3 Evaluation of TALEN-induced mutagenesis efficiencies for *sp7a* in common carp embryos.

Supplementary Figure S4 Evaluation of CRISPR-Cas9-induced mutagenesis efficiencies for *runx2* in common carp embryos.

Supplementary Figure S5 Evaluation of CRISPR-Cas9-induced mutagenesis efficiencies for *opga* in common carp embryos.

Supplementary Figure S6 Evaluation of CRISPR-Cas9-induced mutagenesis efficiencies for *bmp2ab* in common carp embryos.

Supplementary Figure S7 Evaluation of CRISPR-Cas9-induced mutagenesis efficiencies for *sp7b* in common carp embryos.

Supplementary Figure S8 Malformed craniofacial bones of one-month-old *sp7a*-CRISPR mutant common carps shown by Alizarin Red staining.

Supplementary Figure S9 Malformed trunk bones of one-month-old *sp7a*-CRISPR mutant common carps shown by Alizarin Red staining.

Supplementary Figure S10 Malformed scales in two-month-old *sp7a*-CRISPR mutant common carps.

Supplementary Figure S11 Malformed pharyngeal teeth and arches in one-month-old *sp7a*-CRISPR mutant common carps.

Supplementary Figure S12 Indels in the CRISPR-Cas9-targeted *mstnba* site in the testis of three-month-old 5# *mstnba*-CRISPR mutant carp revealed by sequencing.

Supplementary Figure S13 Orginal images of gels and blots.

Supplementary Table S1 Toxicities of TALEN mRNAs and gRNA-Cas9 nucleases in zebrafish embryos

Gene of common carp	Nuclease platform	Dosage of injected RNA (ng/ul)	The ratio of normal (%)	The ratio of dead (%)	The ratio of deformed (%)
runx2	TALEN	Each arm:250	50.3	22.2	27.5
sp7a	TALEN	Each arm:250	61.4	17.5	21.1
mstnba	CRISPR/Cas9	Cas9:300;gRNA:25	62.9	31.4	5.7
mstnba	CRISPR/Cas9	Cas9:300;gRNA:50	82.3	14.1	3.0
mstnba	CRISPR/Cas9	Cas9:300;gRNA:100	80.0	8.3	11.7
sp7a	CRISPR/Cas9	Cas9:300;gRNA:50	63.0	22.2	14.8
sp7a	CRISPR/Cas9	Cas9:300;gRNA:100	88.3	2.6	9.1

Supplementary Table S2 TALEN- or CRISPR-Cas9-induced mutagenesis efficiencies evaluated in zebrafish embryos

Gene of common carp	Nuclease platform	Dosage of injected Cas9/Talen mRNA	Dosage of injected gRNA	Dosage of injected purified plasmid	Mutation frequency	Mutation type
runx2	TALEN	250 ng/ul of each arm		50 ng/ul	2.13%	deletion
sp7a	TALEN	250 ng/ul of each arm		50 ng/ul	2.5%	insertion
mstnba	CRISPR-Cas 9	300 ng/ul Cas9	25/50/100 ng/ul	100 ng/ul	21.0%/10.8%/ 25.4%	deletion
sp7a	CRISPR-Cas 9	300 ng/ul Cas9	50/100 ng/ul	100 ng/ul	5%	deletion

Supplementary Table S3 Mutation rates of each gene using the TALEN system in the common carp embryos at 48-72 hpf

Gene	T7E1（%）	Enzyme digestion（%）	Sequencing（%）
runx2	15.2	1.23	5
sp7a	36.8		20
mstnba	29.1	13.2	27.3
spp1a	81.5		75

Supplementary Table S4 Comparisons of mutation rates using the TALEN system based upon the spacer length

Gene knockout using TALEN	***spp1a***	***sp7a***	***mstnba***	***runx2***
**Spacer length**	15	23	28	28
**Mutation rate**	75%	20%	27.3%	5%

Supplementary Table S5 Mutation rates of each gene using the CRISPR-Cas9 system in common carp embryos at 48-72 hpf

Gene	Dosage of injected RNA (ng/ul)	T7E1（%）	Enzyme digestion（%）	Sequencing（%）
runx2	50/100	43.7	55.9/65.7	66.7
sp7a	100/150/200	66.4/81.8/70.1	93.5/99.1/92.8	100
mstnba	25/50/100	70.0/71.5/68.0	80.7/81.9/76.8	60
bmp2ab	100	40.2	76.2	30
opga	100	84.1	53.1	100
sp7b	100	41.2	52.8	50

Supplementary Table S6 PCR primers

Gene		Forward primer (5' - 3')	Reverse primer (5' - 3')	Amplicon size (bp)	Expected endonuclease fragments (bp)
runx2	TALEN	TGTGTCCGTTTCTGTTCAGG	AAGGCTACACAGAAGCCATGT	411	287/124
sp7a	TALEN	GCTGGTGCATATTTCGGTCT	CTGGAGGTTTGGAAACTGGA	442	
mstnba	TALEN	ACAACACTGCAAGCATTTGTG	ACATCTGCAACTGATGGTCAA	400	276/126
spp1a	TALEN	CGTTGTCCCCTCCACAGTAG	AACAGTCTCACTTAGGATGAC	463	
runx2	CRISPR-Cas9	TTTCAATCGGCTGAGTTCCT	GTGCCACATCATTCATCTGC	305	218/87
sp7a	CRISPR-Cas9	CTGACCAGCGTCAACACCTA	CCGGGTTTAGAGACGTGAAA	256	185/71
mstnba	CRISPR-Cas9	GCATTTGTGACACCTGGAGA	CTGTGGCCATGGTAATGATG	271	165/96/10
bmp2ab	CRISPR-Cas9	GGACTCAAACACAGACCGAGT	CCTGTCAGACACGCAGGATA	278	214/50
opga	CRISPR-Cas9	GTATTGCTTTTGCCGGTTCT	GTCCGGGATGTAGTTCCAGA	210	176/34
sp7b	CRISPR-Cas9	AACCTGTCGCTTCTCAAGGA	AGCCTTGCCATAAACCTTCC	368	238/130
runx2	gRNA	GATCACTAATACGACTCACTATAGGTCACAGTGGACGGACCGC GTTTTAGAGCTAGAAA	AAAAGCACCGACTCGGTGCC	125	
sp7a	gRNA	GATCACTAATACGACTCACTATAGGGCATCCATCCCGGAATCAG TTTTAGAGCTAGAAA	AAAAGCACCGACTCGGTGCC	125	
mstnba	gRNA	GATCACTAATACGACTCACTATAGGTCAAGCTACTCTTACCCAGT TTTAGAGCTAGAAA	AAAAGCACCGACTCGGTGCC	125	
bmp2ab	gRNA	GATCACTAATACGACTCACTATAGGTGGTTCCGCAATACATGAG TTTTAGAGCTAGAAA	AAAAGCACCGACTCGGTGCC	125	
opga	gRNA	GATCACTAATACGACTCACTATAGGTTCTGTCTGGAGCCGGAGG	AAAAGCACCGACTCGGTGCC	125	

sp7b	gRNA	TTTTAGAGCTAGAAA<br>GATCACTAATACGACTCACTATAGGGGCTTGTACATGTCCTGAG<br>TTTTAGAGCTAGAAA	AAAAGCACCGACTCGGTGCC	125
mstnaa	RT-PCR	AGGTTCTTGAAACAACCAAGC	AAGGAACGAAACAGCCACAG	232
mstnba	RT-PCR	GCAATGACCTGGCTGTGAC	AGACCCAGATTTCTCCGTGA	274
mstnbb	RT-PCR	TTGCAACCCTTTCTGGAGGT	GGATACTTCTGCACACATTC	197
mstnab	RT-PCR	CCATCACATTCAGCTGGGTT	GGAGATGAGAGTGGTGCTTA	290
β-actin1a	qRT-PCR	TGTCTTCCCCTCCATTGTTG	GGTCACAATACCGTGCTCAA	148
myoda	qRT-PCR	GGCGTGTCGTTGAAGTATGA	AACTGCTCTGATGGCATGGT	95
myf5a	qRT-PCR	GCCATACAGGACTGTTGCAG	CAGGATCTCCTCAGGGAACA	91
myogenina	qRT-PCR	GGTTTGCTCTGAGGATTCGT	AGGCACTGGTCAGCTCTCTC	125

Figure S1

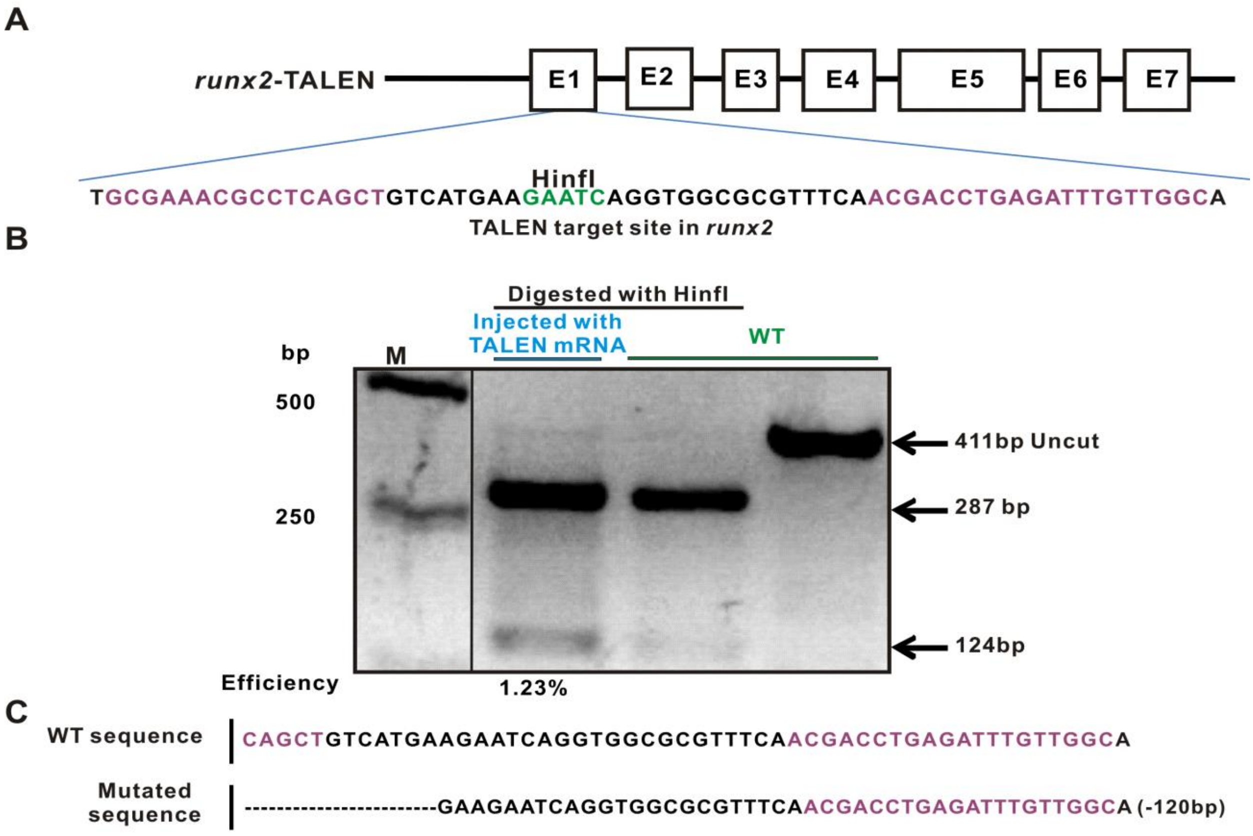

Supplementary Figure S1 Evaluation of TALEN-induced mutagenesis efficiencies for *runx2* in common carp embryos. (A) Diagram of TALEN-targeting the first exon of common carp *runx2*. The left and right arms of common carp *runx2* are highlighted in purple. The restriction enzyme HinfI site inside the targeted fragment is highlighted in green. E, exon. (B) Enzymatic digestion analysis of TALEN-mediated cleavage at common carp *runx2*. PCR products were digested with HinfI. Uncut (411 bp) and cut PCR products (287 bp and 124 bp) are indicated. Capped mRNAs of two *runx2* TALEN arms were co-microinjected into one- or two-cell common carp embryos at a concentration of 250 pg each. Estimated mutagenesis efficiency of 1.23% is indicated at the bottom. WT, wild type; M, marker. (C) A representative type of deletion mutation (-120 bp) in the *runx2* TALEN target site, revealed by DNA sequencing analysis.

Figure S2

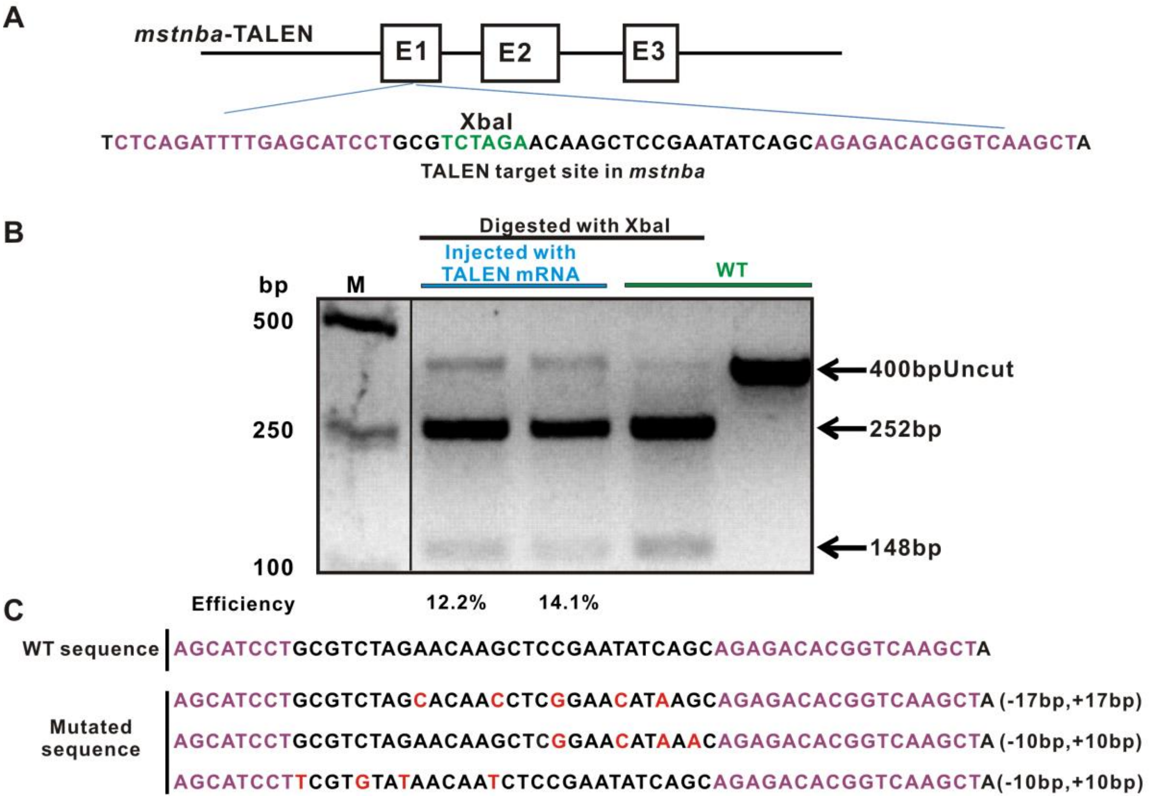

Supplementary Figure S2 Evaluation of TALEN-induced mutagenesis efficiencies for *mstnba* in common carp embryos. (A) Diagram of TALEN-targeting the first exon of common carp *mstnba*. The left and right arms of *mstnba* TALEN are highlighted in purple, and the restriction enzyme XbaI is highlighted in green. E, exon. (B) Enzymatic digestion analysis of TALEN-mediated cleavage at common carp *mstnba*. PCR products were digested with XbaI. Uncut (400 bp) and cut PCR products (252 bp and 148 bp) are indicated. Capped mRNAs of two *runx2* TALEN arms were co-microinjected into one- or two-cell common carp embryos at a concentration of 250 pg. Mutagenesis efficiencies were estimated to 12.2% and 14.1% (at the bottom). WT, wild type; M, marker. (C) Mutations in common carp *mstnba*, revealed by sequencing analysis. Three types of mutations are detected in the *mstnba* TALEN targeted site. Substitutions in the spacer are in red.

Figure S3

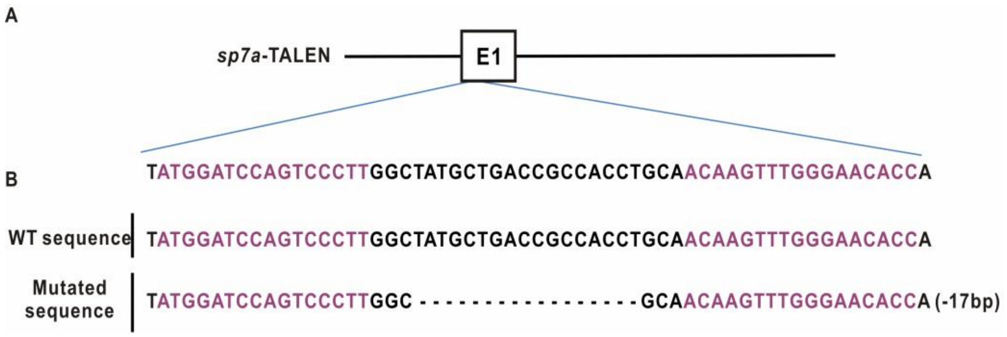

Supplementary Figure S3 Evaluation of TALEN-induced mutagenesis efficiencies for *sp7a* in common carp embryos. (A) Diagram of TALEN targeting the first exon of common carp *sp7a*. The left and right arms of common carp *sp7a* TALEN are highlighted in purple. (B) One representative type of deletion mutation (-17) in *sp7a* TALEN targeted site, revealed by DNA sequencing analysis.

Figure S4

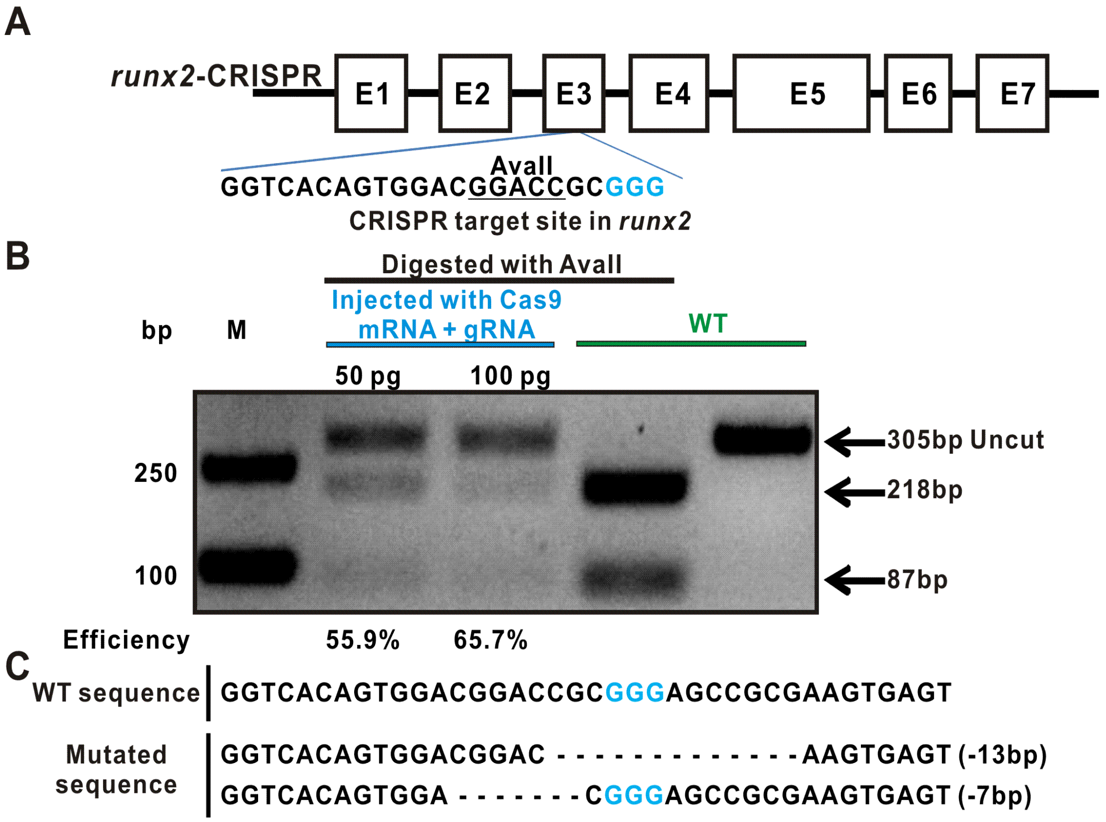

Supplementary Figure S4 Evaluation of CRISPR-Cas9-induced mutagenesis efficiencies for *runx2* in common carp embryos. (A) Schematic of gRNA-Cas9 targeting the third exon of common carp *runx2*. The restriction enzyme AvaII site inside the targeted fragment is underlined. The PAM sequences are labeled in blue. E, exon. (B) Enzymatic digestion analysis of gRNA-Cas9-mediated cleavage at common carp *runx2*. 50 pg and 100 pg of *runx2* Cas9 gRNA along with 300 pg of Cas9 mRNAs were microinjected into one- or two-cell common carp embryos (below the blue line), respectively. PCR products were digested with AvaII. Uncut (305 bp) and cut (218 bp and 87 bp) bands are indicated. WT, wild type; M, marker. Mutagenesis efficiencies were estimated to be 55.9% for the 50 pg group, and 65.7% for the100 pg group, respectively. (C) Types of *runx2* mutations in F_0, revealed by DNA sequencing analysis.

Figure S5

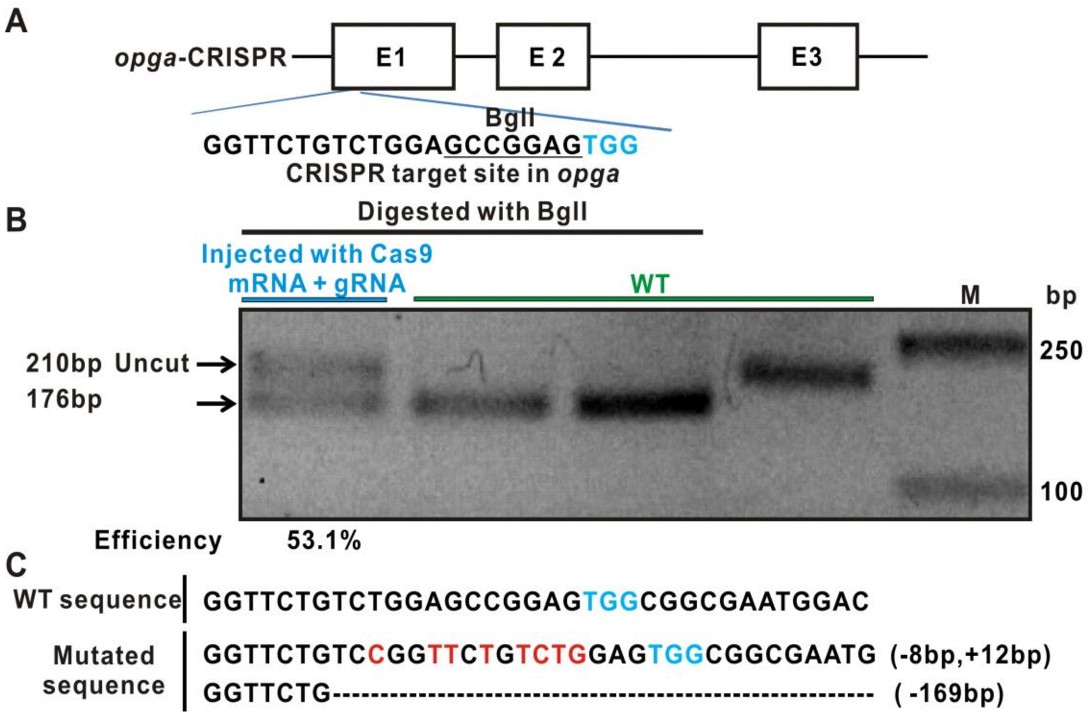

Supplementary Figure S5 Evaluation of CRISPR-Cas9-induced mutagensis efficiencies for *opga* in common carp embryos. (A) Schematic of gRNA-Cas9 targeting the first exon of common carp *opga*. The restriction enzyme BglI site located inside the targeted fragment is underlined. The PAM sequences are labeled in blue. (B) Enzymatic digestion analysis of gRNA-Cas9-mediated cleavage at common carp *opga*. 100 pg of *opga* Cas9 gRNA along with 300 pg of Cas9 mRNAs were microinjected into one- or two-cell common carp embryos (below the blue line). PCR products were digested with BglI. Uncut (210 bp) and cut (176 bp and 34 bp) bands are indicated. WT, wild type; M, marker. Mutagenesis efficiency was estimated to be 53.1%. (C) Types of common carp *opga* mutations revealed by DNA sequencing analysis. Insertions in the targeted fragment are in red.

Figure S6

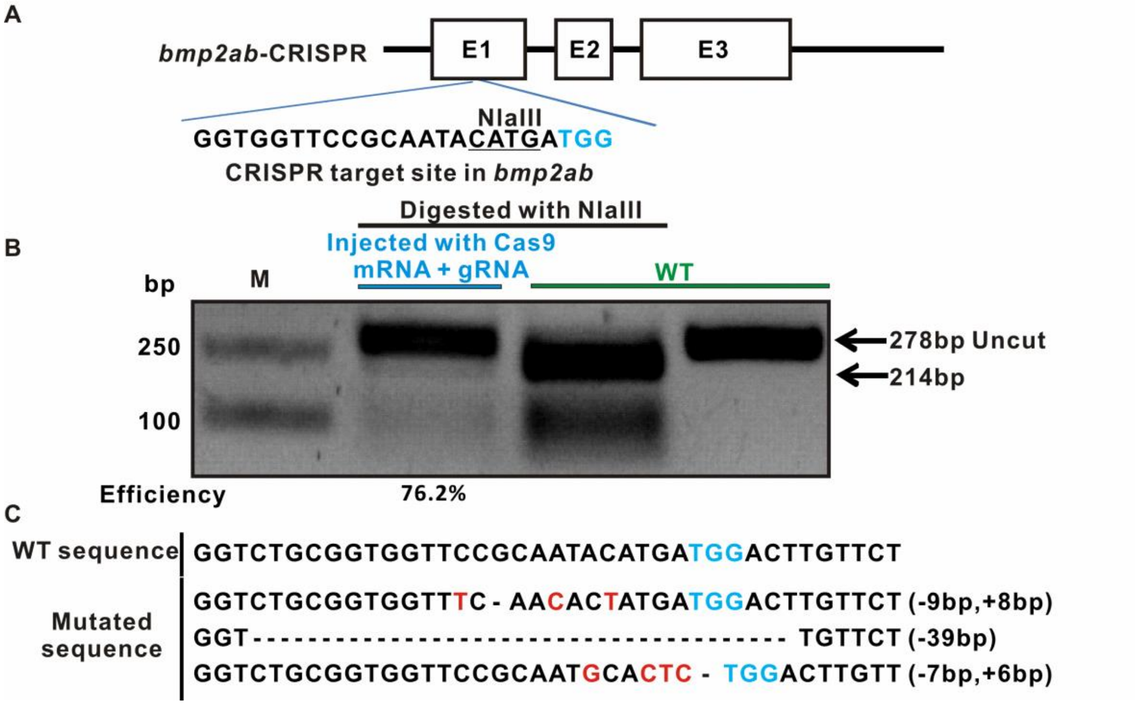

Supplementary Figure S6 Evaluation of CRISPR-Cas9-induced mutagenesis efficiencies for *bmp2ab* in common carp embryos. (A) Schematic of gRNA-Cas9 targeting the first exon of common carp *bmp2ab*. The restriction enzyme NlaIII site inside the targeted fragment is underlined. The PAM sequences are labeled in blue. E, exon. (B) Enzymatic digestion analysis of gRNA-Cas9-mediated cleavage at common carp *bmp2ab*. 100 pg of *bmp2ab* Cas9 gRNA along with 300 pg of Cas9 mRNAs were microinjected into one- or two-cell common carp embryos (below the blue line). PCR products were digested with NlaIII. Uncut (278 bp) and cut (214 bp and 64 bp) bands are indicated. WT, wild type; M, marker. Mutagenesis efficiency was estimated to be 76.2%. (C) Types of *bmp2ab* mutations in F_0 revealed by DNA sequencing analysis. Insertions in the targeted fragment are in red.

Figure S7

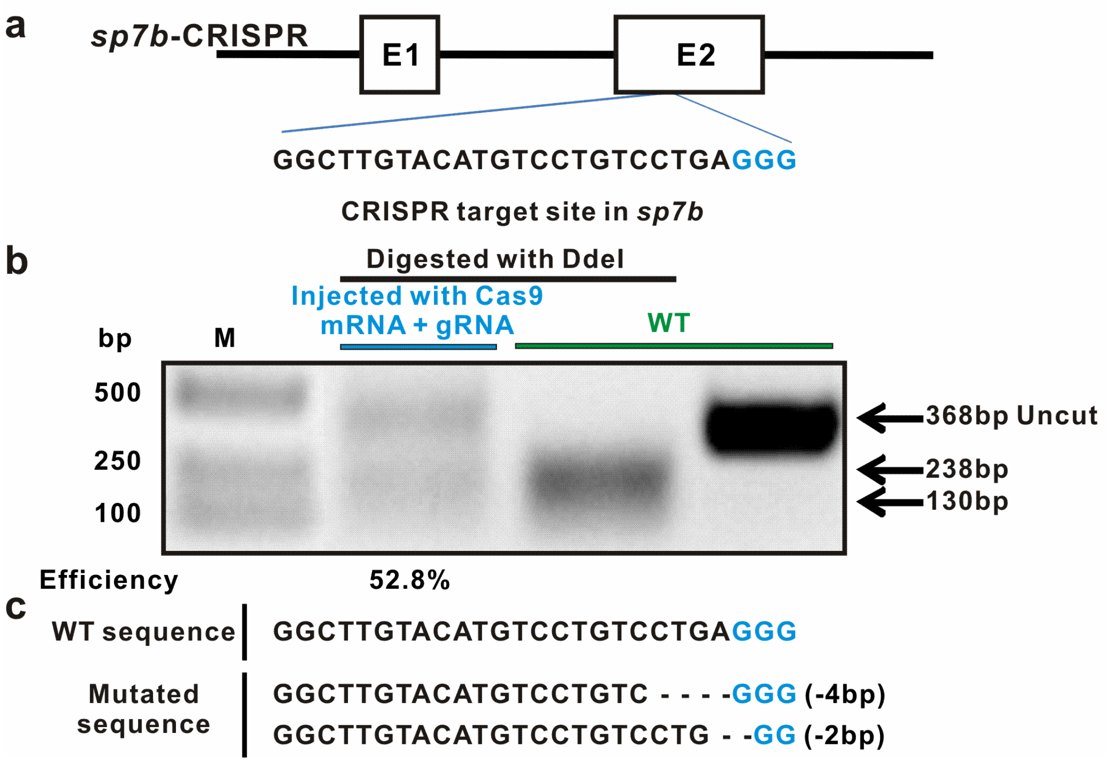

Supplementary Figure S7 Evaluation of CRISPR-Cas9-induced mutagenesis efficiencies for *sp7b* in common carp embryos. (A) Schematic of gRNA-Cas9 targeting the second exon of common carp *sp7b*. The restriction enzyme DdeI site inside the targeted fragment is underlined. The PAM sequences are labeled in blue. E, exon. (B) Enzymatic digestion analysis of gRNA-Cas9-mediated cleavage at common carp *sp7b*. 100 pg *sp7b* Cas9 gRNA along with 300 pg of Cas9 mRNAs were microinjected into one- or two-cell common carp embryos (below the blue line). PCR products were digested with DdeI. Uncut (368 bp) and cut (238 bp and 130 bp) bands are indicated. WT, wild type; M, marker. Mutagenesis efficiency was estimated to be 52.8%. (C) Types of *sp7b* mutations in F_0 revealed by DNA sequencing analysis.

Figure S8

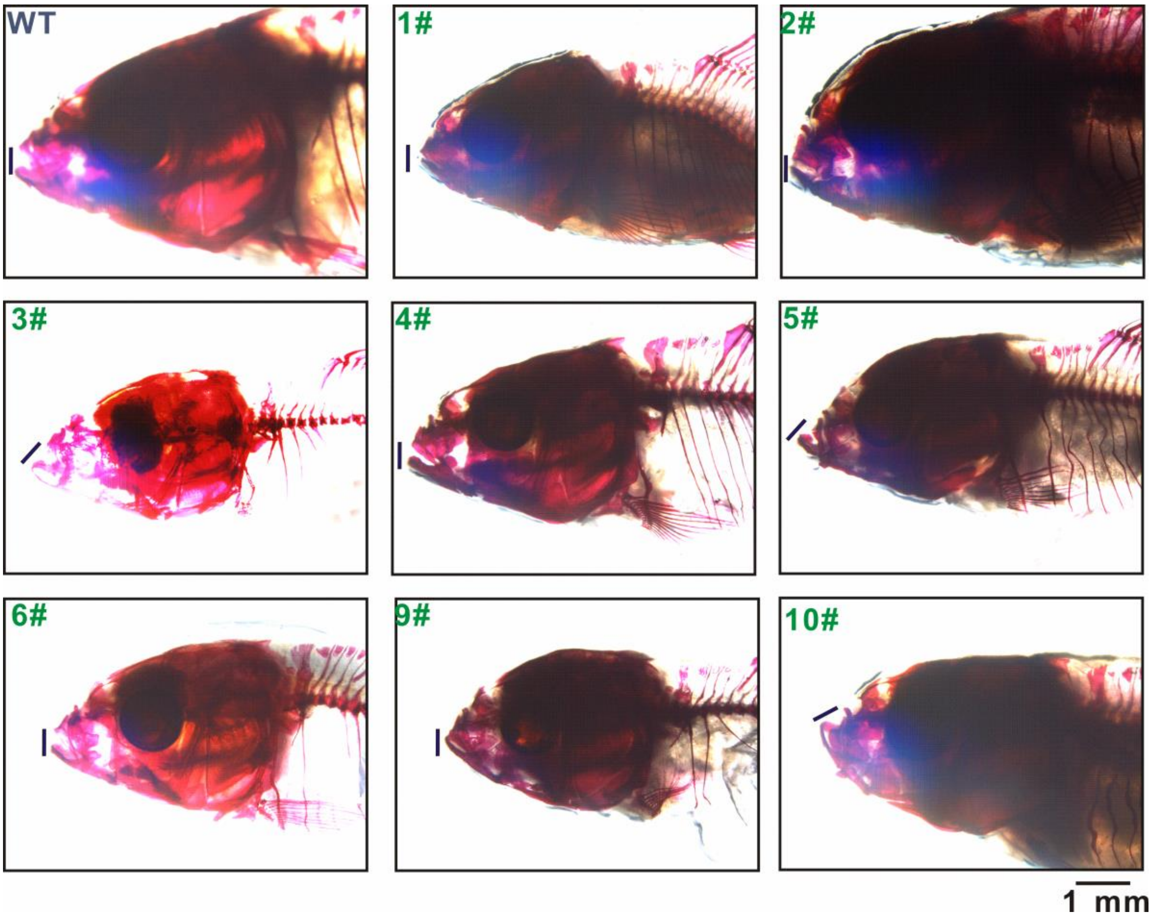

Supplementary Figure S8 Malformed craniofacial bones of one-month-old *sp7a*-CRISPR mutant common carps shown by Alizarin Red staining. Blue lines represent the angle between the maxilla and the premaxilla. Three out of the eight fishes display maxilla insufficiency. Numbers correspond to the carps in Fig.6A. WT, Wild type. Scale bar: 1 mm.

Figure S9

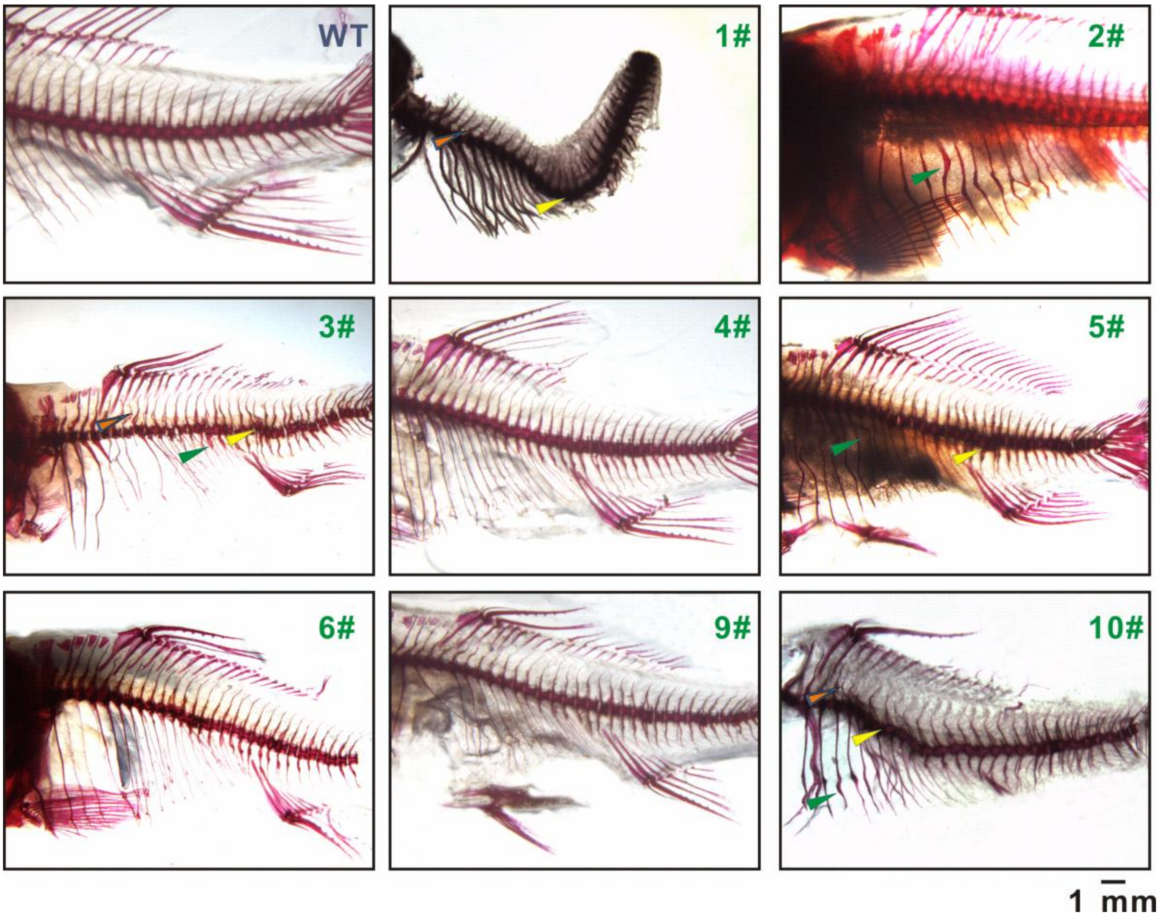

Supplementary Figure S9 Malformed trunk bones of one-month-old *sp7a*-CRISPR mutant common carps shown by Alizarin Red staining. Three out of the eight fishes display curved hemal spines (orange arrowheads). Four out of the eight fishes display crinkled neural spines (green arrowheads) and deformed centrums (yellow arrowheads). Numbers correspond to carps in Fig. 6A. WT, Wild type. Scale bar: 1 mm.

Figure S10

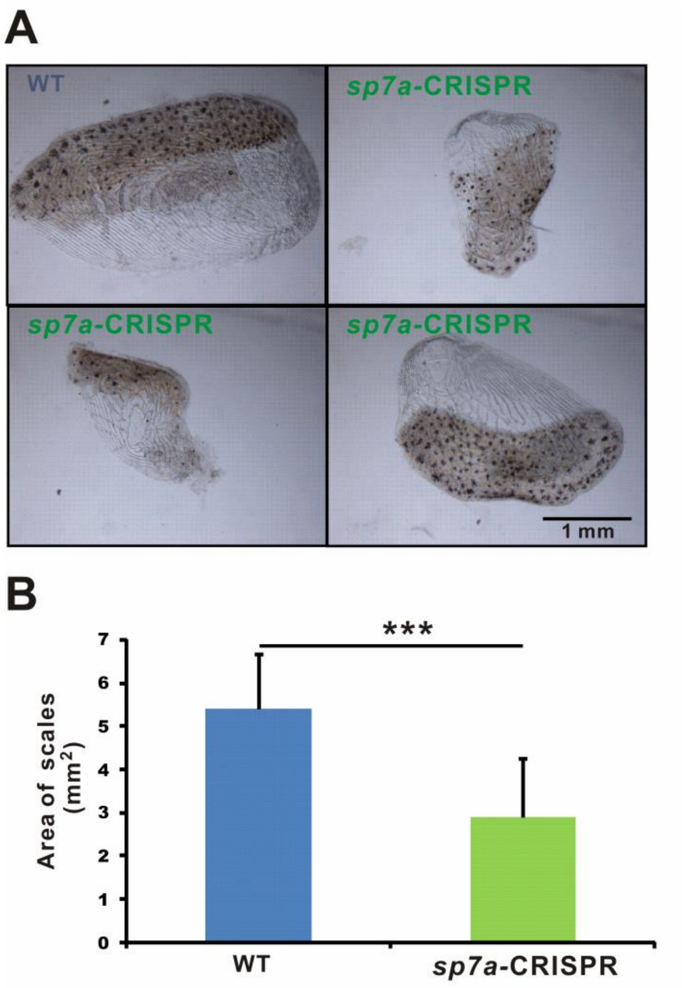

Supplementary Figure S10 Malformed scales in two-month-old *sp7a*-CRISPR mutant common carps. (A) Small-sized and irregular-shaped scales in *sp7a* -CRISPR mutant carps, shown by bright-field photos. Scales were from three wild-type and three *sp7a* -CRISPR carps (11#, 12# and 13#). Ten scales of each group were randomly selected for photographing and estimating the scale area. Scale bar: 1 mm. (B) Significantly reduced scale areas in *sp7a*-CRISPR carps. Values are means ±SD. Two-tailed Student's *t*-test was conducted, $P<0.001$.

Figure S11

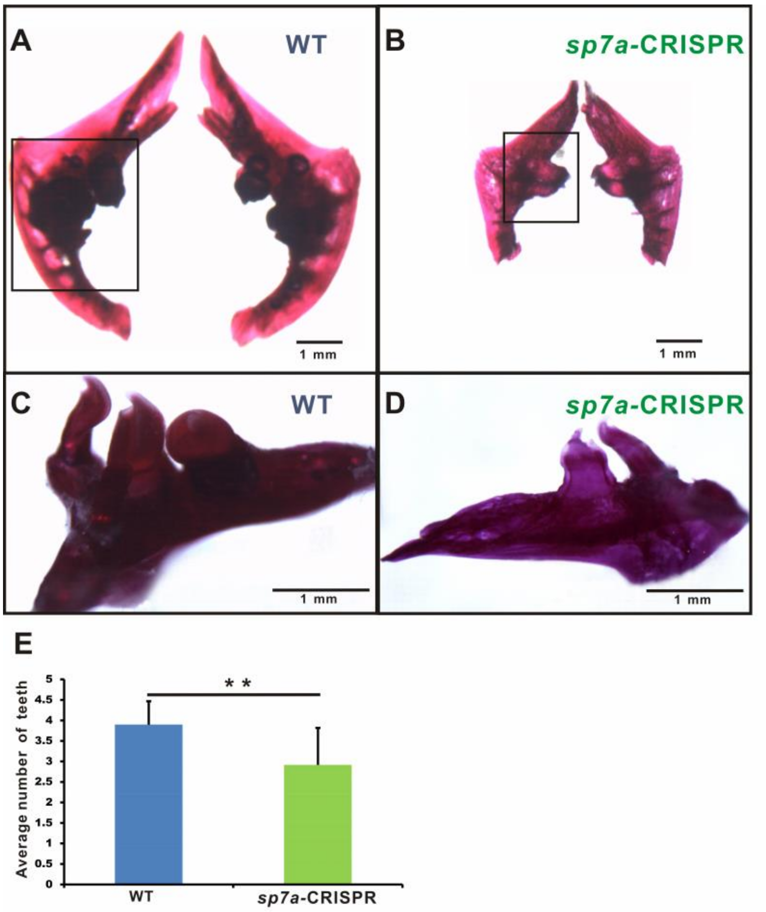

Supplementary Figure S11 Malformed pharyngeal teeth and arches in one-month-old *sp7a*-CRISPR mutant common carps. (A, B) Small-sized pharyngeal arches in *sp7a*-CRISPR 3# (B) carp in comparison with wild-type control (A), shown by Alizarin Red staining. (C, D) High magnification of the rectangle frame in Supplementary Figs. S11A (C) and S11B (D). Scale bar: 1 mm. (E) Fewer teeth in one-month-old *sp7a*-CRISPR mutant common carps. Values are means ±SD. Two-tailed Student's *t*-test was conducted, P=0.007. Pharyngeal teeth were counted from three wild-type and eight *sp7a*-CRISPR carps (1# to 06#, 9# and10# in Fig. 6A).

Figure S12

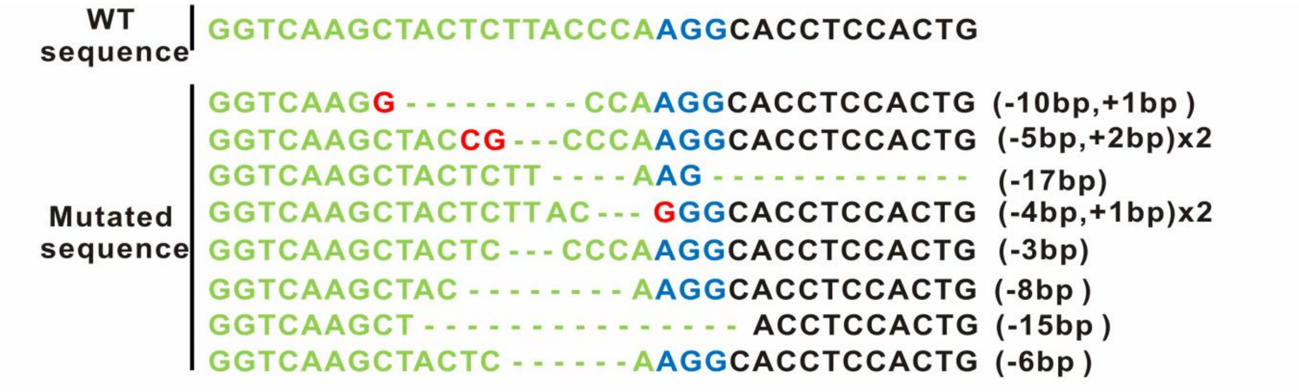

Supplementary Figure S12 Indels in the CRISPR-Cas9-targeted *mstnba* site in the testis of three-month-old 5# *mstnba*-CRISPR mutant carp revealed by sequencing. The gRNA-targeted sequence is highlighted in green, and the PAM sequences are labeled in blue. Insertions in the targeted fragment are in red.

Figure S13

A

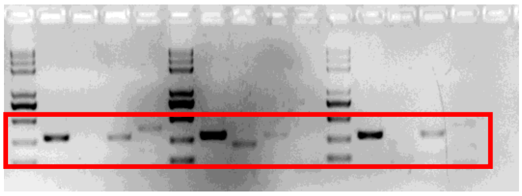

B

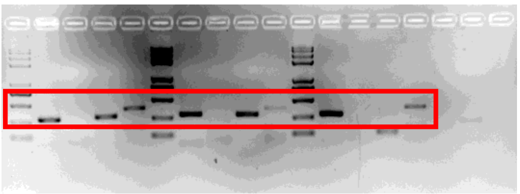

C

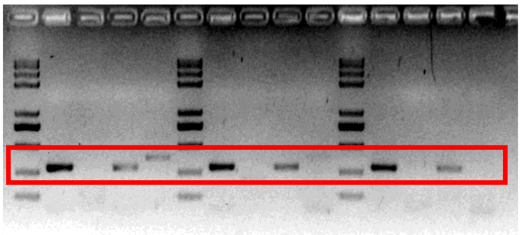

D

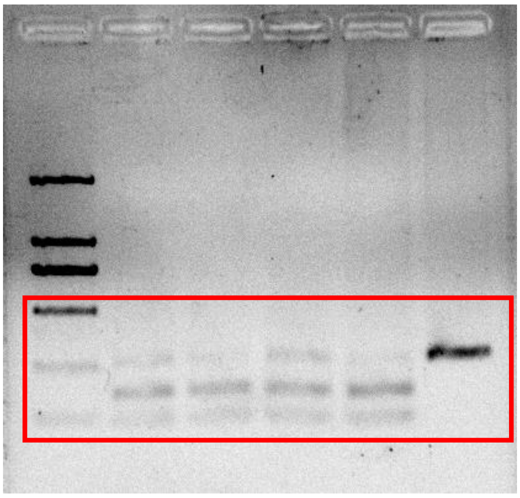

E

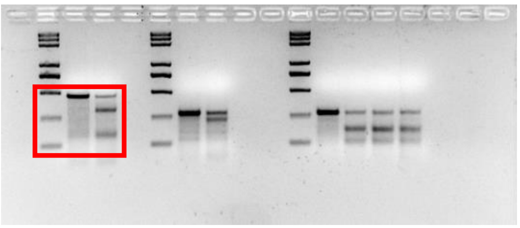

F

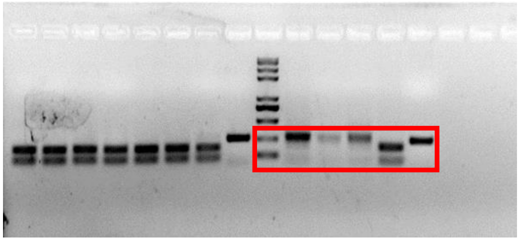

G

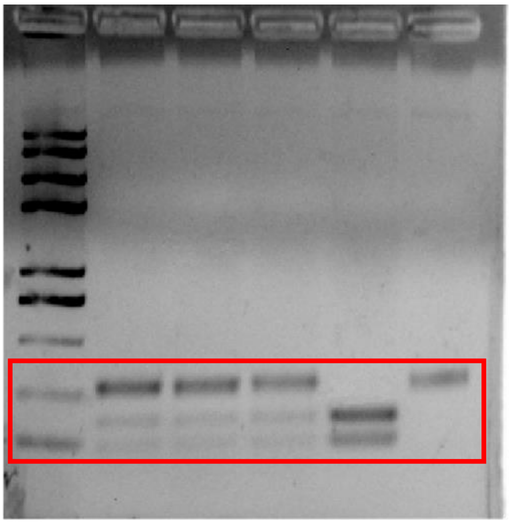

H

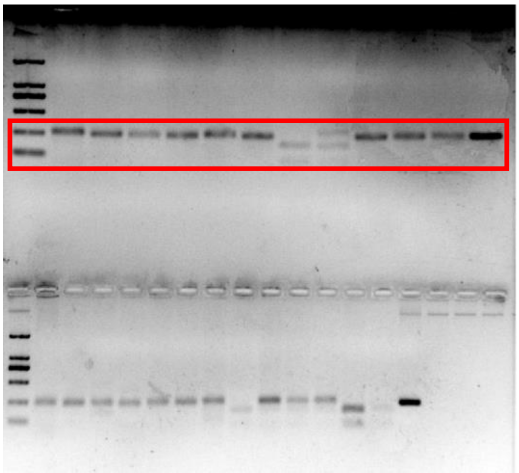

I

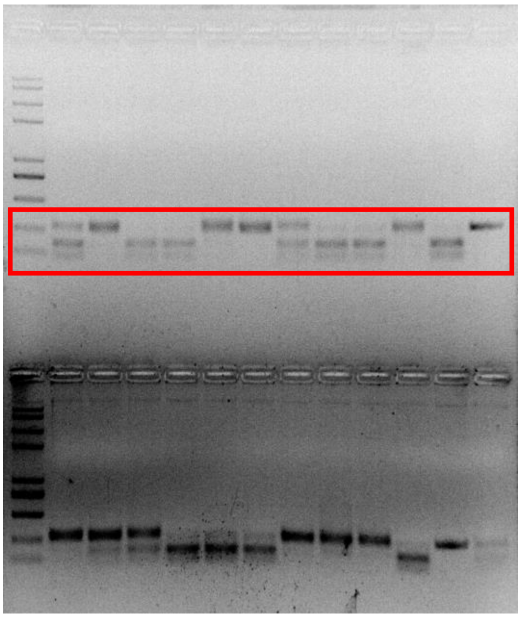

J

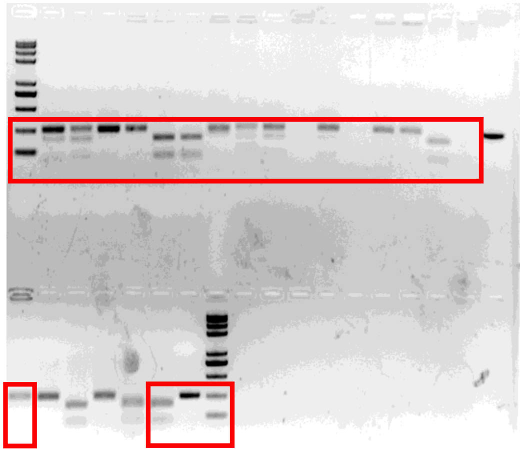

K

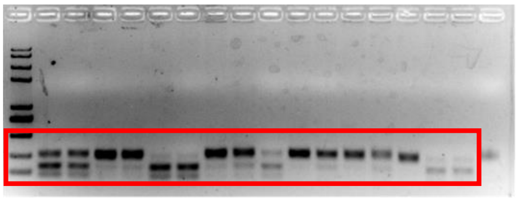

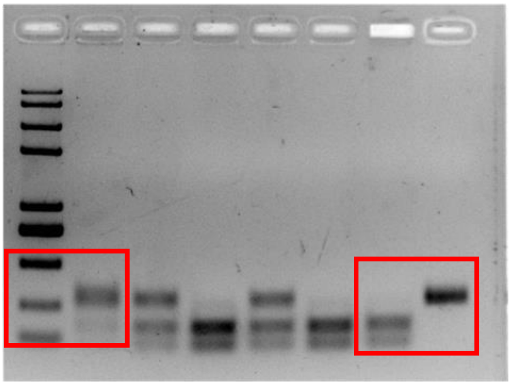

L

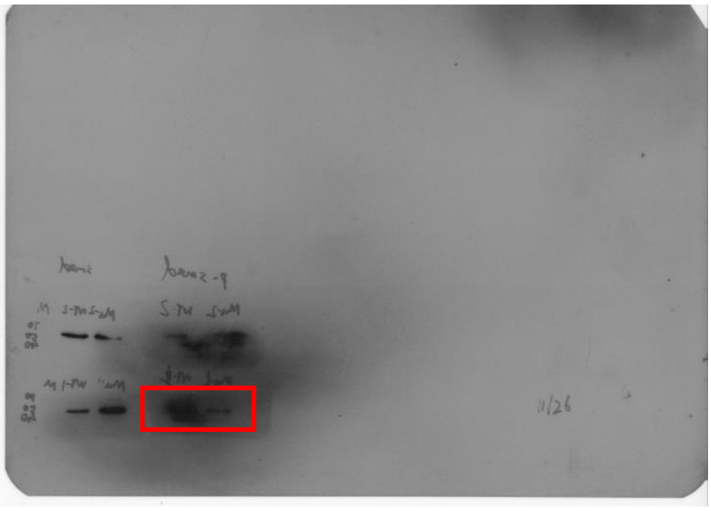

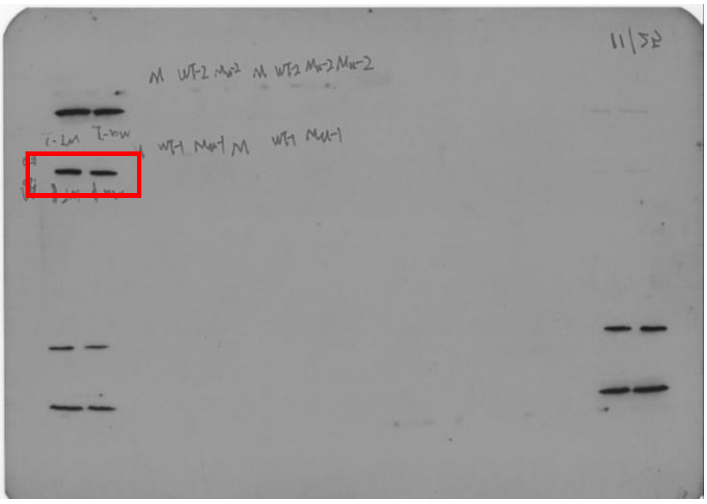

Supplementary Figure S13 Original images of gels and blots.

RT-PCR results in Fig. 1C (A), Fig. 1D (B) and Fig. 1E (C) were shown. Enzyme digestion results in Fig. 2C (D), Fig. 4B (F), Fig. 4E (G), Fig. 6A (H), Fig. 5A (I), Fig. 7A (J), Fig. 7B (K) are shown. (E) T7E1 result in Fig. 3C was shown, (L) Western blotting result in Fig. 5E was shown. Red lines represent cropped lines.
